# Microglial APOE3 Christchurch protects neurons from Tau pathology in a human iPSC-based model of Alzheimer’s disease

**DOI:** 10.1016/j.celrep.2024.114982

**Published:** 2024-11-28

**Authors:** Guoqiang George Sun, Cheng Wang, Randall C. Mazzarino, Paula Andrea Perez-Corredor, Hayk Davtyan, Mathew Blurton-Jones, Francisco Lopera, Joseph F. Arboleda-Velasquez, Yanhong Shi

**Affiliations:** 1 Department of Neurodegenerative Diseases, Beckman Research Institute of City of Hope, 1500 E. Duarte Road, Duarte, CA 91010, USA; 2 Schepens Eye Research Institute of Mass Eye and Ear and Department of Ophthalmology, Harvard Medical School, Boston, MA 02114, USA; 3 Department of Neurobiology & Behavior, Institute for Memory Impairments & Neurological Disorders and Sue & Bill Gross Stem Cell Research Center, University of California Irvine, Irvine, CA 92697, USA; 4 Grupo de Neurociencias de la Universidad de Antioquia, Medellin 050010, Colombia; 5 These authors contributed equally; 6 Lead contact; 7 Francisco Lopera is deceased

## Abstract

Alzheimer’s disease (AD) is the most prevalent neurodegenerative disorder characterized by extracellular amyloid plaques and neuronal Tau tangles. A recent study found that the APOE3 Christchurch (APOECh) variant could delay AD progression. However, the underlying mechanisms remain unclear. In this study, we established neuron-microglia co-cultures and neuroimmune organoids using isogenic APOE3 and APOECh microglia derived from human induced pluripotent stem cells (hiPSCs) with PSEN1 mutant neurons or brain organoids. We show that APOECh microglia are resistant to Aβ-induced lipid peroxidation and ferroptosis and therefore preserve the phagocytic activity and promote pTau clearance, providing mechanistic insights into the neuroprotective role of APOE3Ch microglia. Moreover, we show that an APOE mimetic peptide can mimic the protective effects of APOECh microglia. These findings demonstrate that the APOECh microglia plays a causal role in microglial neuroprotection, which can be exploited for therapeutic development for AD.

## In brief

Sun et al. demonstrate that APOE3 Christchurch microglia protect neurons from Aβ-induced Tau pathology by regulating lipid peroxidation and phagocytosis. Targeting APOE and lipid metabolism in microglia represents a promising therapeutic strategy for Alzheimer’s disease.

## Graphical Abstract

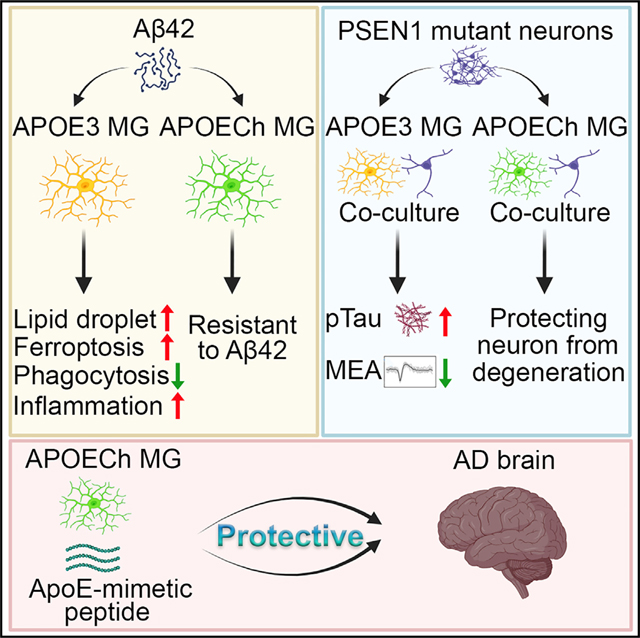

## INTRODUCTION

Alzheimer’s disease (AD) is a devastating neurodegenerative disease.^1,2^ However, there is little progress toward therapeutic development for AD. Although the US Food and Drug Administration (FDA) has approved aducanumab and Leqembi as disease-modifying treatments for AD by targeting β-amyloid (Aβ), their impact on long-term disease progression remains to be watched.^3,4^ Therefore, alternative strategies are still needed.

Familial AD (fAD) is a rare form of AD that is primarily caused by genetic mutations in three genes: the amyloid precursor protein (*APP*) gene, presenilin 1 (*PSEN1*) gene, and presenilin 2 (*PSEN2*) gene.^5–10^ Mutations of *PSEN1* and *PSEN2* genes affect the function of γ-secretase complex, which is responsible for the cleavage of the APP protein and releasing Aβ peptides, leading to increased ratio of Aβ42/Aβ40, which contributes to the formation of amyloid plaques. These plaques, in conjunction with other contributing factors, initiate a cascade of neurodegenerative events that lead to clinical manifestations of AD.^11–14^

Apolipoprotein E (APOE) is a major cholesterol carrier that mediates lipid transport in the brain.^15^ There are three major isoforms of APOE, namely APOE2, APOE3, and APOE4. The ε4 allele of APOE is the strongest genetic risk factor for AD.^16,17^ In addition to the common variants, several rare variants of APOE have been identified, including the APOE Christchurch (APOECh) variant (R136S),^18^ the APOE3-Jacksonville variant (V236E),^19^ and the APOE4 R251G variant.^20^ Recently, researchers found that an individual harboring the PSEN1 E280A mutation from the Paisa PSEN1 E280A kindred showed delayed onset of AD as shown by the limited development of Tau pathology despite the presence of excess amyloid plaques. Subsequent whole-exome sequencing revealed that this individual carried the homozygous APOE3 Christchurch (Ch) variant, suggesting a protective role for this rare APOE variant in AD pathogenesis.^21^ However, whether the APOECh variant is causal to the delayed onset of AD and what the underlying mechanism is remain to be determined.

Since the advent of induced pluripotent stem cell (iPSC) technology,^22,23^ human iPSCs (hiPSCs) have been rapidly applied to disease modeling,^24,25^ especially modeling neurological diseases for which primary human brain tissues are not easily accessible.^26,27^ Cells and organoids derived from hiPSCs have been used to model various aspects of AD.^28–37^

Here, we investigate the neuroprotective effects of the APOECh variant using hiPSC-based models. We developed both 2D neuron-microglia (N-MG) co-culture and 3D neuroimmune organoidmodels using isogenic APOE3 and APOECh microglia derived from parental or CRISPR-Cas9-edited hiPSCs together with hiPSC-derived PSEN1 mutant neurons or brain organoids. The N-MG co-cultures were exposed to postmortem brain-derived materials from patients with AD. RNA sequencing (RNA-seq) of isogenic APOE3 and APOECh microglia isolated from N-MG co-cultures were performed to identify pathways of differentially expressed genes to provide insights into mechanisms underlying the neuroprotective effects of the APOECh variant.

## RESULTS

### APOECh microglia preserve phagocytic function in co-cultures with PSEN1 mutant neurons

**Figure 1.**
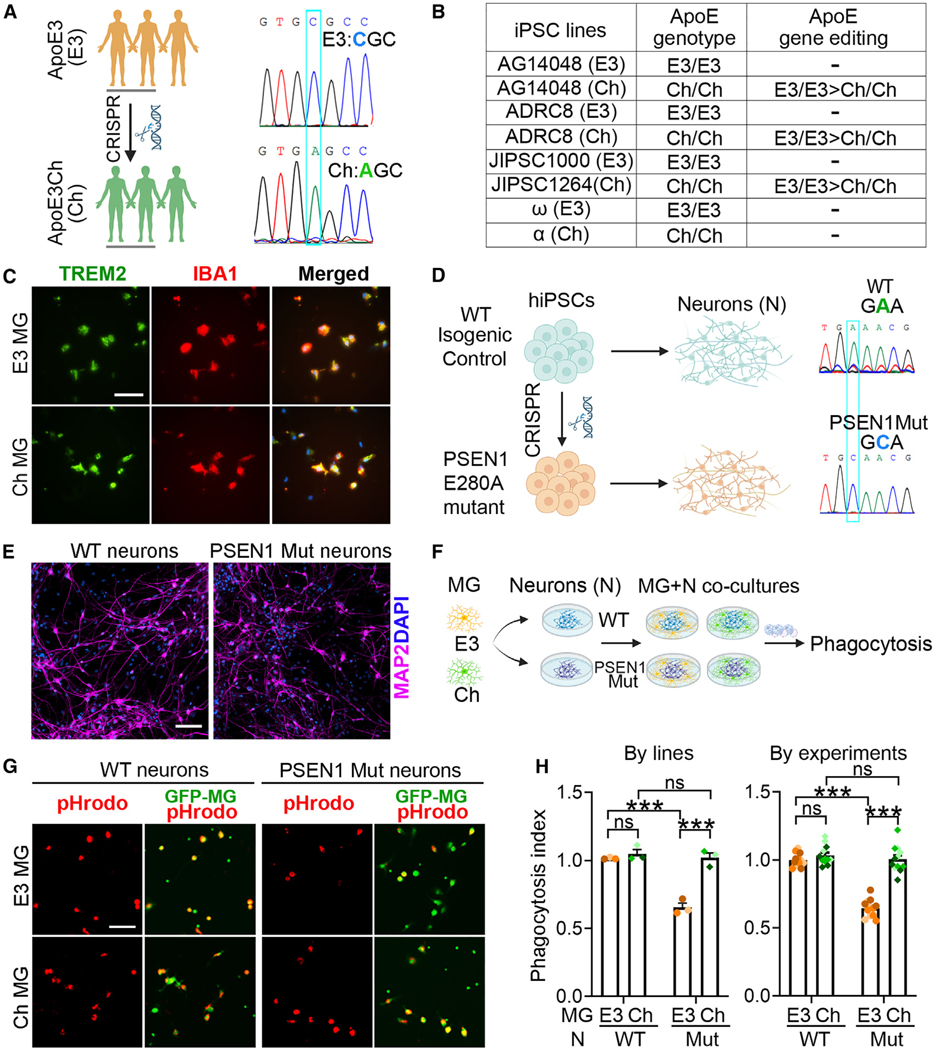
APOECh MG preserve phagocytotic function when co-cultured with PSEN1 mutant neurons (A) A schematic showing APOE3 (E3) and Ch iPSC derivation. Three isogenic Ch iPSC lines were generated from three E3 iPSC lines by CRISPR-Cas9 editing. Sanger sequencing confirmed the APOE genotype in the E3 and Ch iPSCs. (B) A table showing E3 and Ch iPSC lines used in this study. In addition to three pairs of isogenic E3 and Ch iPSCs, one pair of iPSCs with E3 or Ch genotype derived from the Colombian kindred was also included in this study. (C) Representative images of TREM2 and IBA1 staining of iPSC-derived E3 and Ch MG at day 38 post differentiation. Scale bar: 20 μm. (D) A schematic for generation of PSEN1 E280A mutant iPSCs (PSEN1 Mut) by CRISPR-Cas9 editing of the wild-type (WT) iPSCs and the differentiation of iPSCs into neurons. Sanger sequencing confirmed the PSEN1 genotype in the parental and PSEN1 mutant iPSCs. (E) Representative images of MAP2 staining of iPSC-derived neurons at week 4 post differentiation. Scale bar: 100 μm. (F) A schematic illustration of N-MG co-cultures followed by synaptosome phagocytosis. (G) Representative images of phagocytosis of pHrodo-labeled human synaptosome by MG. pHrodo-labeled human synaptosomes were introduced into N-MG co-cultures. MG phagocytosis of the synaptosomes was monitored by live imaging. Scale bar: 50 μm. (H) The phagocytosis index of E3 and Ch MG co-cultured with WT or PSEN1 mutant (Mut) neurons. For the phagocytosis index, we first obtained the percentage of pHrodo-positive MG in total MG, then normalized it using E3 MG co-cultured with WT neurons as the reference. In “by lines,” each dot represents 1 iPSC-MG line. In “by experiments,” each dot represents one image and each color of dots represents one cell line. There are a total of 12 images from three lines with one line showing the same degree of color. Error bars are SEM. ***p < 0.001; ns, *p* > 0.05 by two-way ANOVA followed by Tukey’s multiple comparison test.

To elucidate the role of APOECh variant in the pathogenesis of AD, we generated two isogenic APOECh iPSC lines from two control APOE3 (E3) iPSC lines by CRISPR-Cas9 editing and obtained a pair of isogenic APOECh and APOE3 iPSC lines from the Jackson laboratory (Figures 1A, 1B, and S1; Tables S1 and S2). We also included one APOECh iPSC line from patient α that is homozygous for APOECh and has the PSEN1 E280A mutation corrected to PSEN1 wild type (WT) by CRISPR-Cas9 editing and one APOE3 iPSC line from patient ω that is homozygous for APOE3 and has the PSEN1 E280A mutation corrected to PSEN1 WT (Figure 1B).^38^ APOE is expressed in glial cells including microglia^39^ and important for microglial status and function.^40^ We generated APOECh and APOE3 microglia from four pairs of iPSCs (Figures 1A–1C and S2A) following a published protocol.^41^

To determine if the APOECh variant can protect neurons from the neuropathology induced by the PSEN1 E280A mutation, we generated a PSEN1 E280A mutant iPSC line by CRISPR-Cas9 editing (Figure 1D). Neurons were generated from WT and PSEN1 mutant iPSCs following our published protocol^42^ and expressed the mature neuronal marker MAP2 4 weeks after differentiation (Figure 1E).

As professional phagocytes, microglia play a critical role in clearing various types of debris, including apoptotic neurons, myelin debris, oxidized lipids, and degenerative synapses.^43^ The activity of microglia phagocytosis can be regulated by AD risk genes including APOE.^44^ Because the individual with homozygous APOECh and PSEN1 E280 exhibited limited spread of Tau pathology in the brain,^21^ we hypothesize that APOECh microglia can exhibit enhanced phagocytotic capacity to better clear Tau spreads. To assess the phagocytic activity of APOECh or APOE3 microglia, we co-cultured APOECh or APOE3 microglia with WT or PSEN1 mutant neurons (Figure S2B) and introduced pHrodo-conjugated human fetal brain-derived synaptosomes into the co-cultures (Figure 1F). The pHrodo dye would turn red after being engulfed by microglia. We found a substantial decrease of the phagocytosis index in APOE3 microglia compared to APOECh microglia when co-cultured with PSEN1 mutant neurons but no significant difference in the phagocytosis index between APOECh and APOE3 microglia when co-cultured with WT neurons (Figures 1G and 1H). Moreover, APOE3 microglia co-cultured with PSEN1 mutant neurons exhibited elevated neuroinflammatory response as revealed by a higher induction of IL-1β than that in APOECh microglia co-cultured with PSEN1 mutant neurons (Figure S2C). In conclusion, our observation indicates that APOECh microglia display significantly enhanced phagocytic activity and reduced inflammatory response compared to APOE3 microglia when co-cultured with PSEN1 mutant neurons, suggesting that APOECh microglia may be more resilient to toxic environments and better equipped to maintain their phagocytic function.

### Genes involved in lipid droplet formation, ferroptosis, and phagocytosis pathways are differentially expressed in APOE3 vs. APOECh microglia

**Figure 2.**
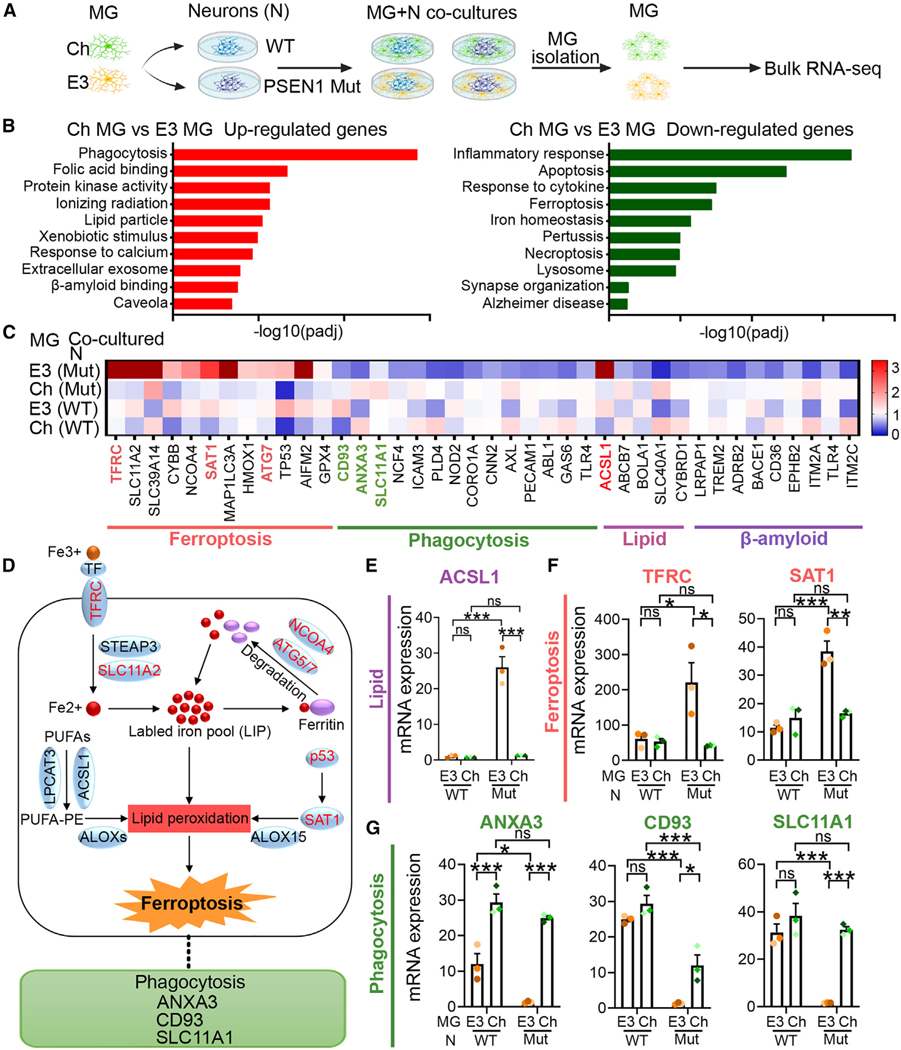
RNA-seq analysis reveals that the ferroptosis pathway is involved in the protection by APOECh (A) A schematic illustration of N-MG co-cultures, and subsequent isolation of MG from the co-cultures for RNA-seq analysis. (B) GO and KEGG analyses of upregulated (left panel) and downregulated (right panel) genes in Ch vs. E3 MG co-cultured with PSEN1 mutant (Mut) neurons, ranked by -log10 (adjusted *p* value). (C) Heatmap of differentially expressed genes (DEGs) in Ch vs. E3 MG (co-cultured with WT or PSEN1 mutant neurons) associated with key pathways in GO and KEGG analysis. (D) Diagram illustrating the pathways and relevant DEGs (shown in red) implicated in ferroptosis. (E) qRT-PCR analysis of the lipid-related gene *ACSL1*. (F) qRT-PCR analysis of ferroptosis-related genes *SAT1* and *TFRC*. (G) qRT-PCR analysis of phagocytosis-related genes *ANXA3*, *CD93*, and *SLC11A1*. *n* = 3 biological repeats for (E)–(G). Error bars are SEM. *p < 0.05, **p < 0.01, ***p < 0.001; ns, *p* > 0.05 by two-way ANOVA followed by Tukey’s multiple comparison test.

To gain insights into the mechanism of APOECh microglia-mediated protection, we prepared RNA from APOECh and APOE3 microglia that had been isolated from the co-culture with either WT or PSEN1 mutant neurons and conducted RNA-seq analysis (Figure 2A). The Gene Ontology (GO) and Kyoto Encyclopedia of Genes and Genomes (KEGG) pathway analysis of differentially expressed genes (DEGs) revealed that the upregulated DEGs are primarily associated with phagocytosis, lipid particles, folic acid, and Aβ binding pathways (Figure 2B), while the downregulated DEGs are mainly related to inflammatory response, apoptosis, ferroptosis, ion homeostasis, and AD pathways (Figure 2B).

Given the critical role of APOE in lipid metabolism, we focused on lipid-related pathways including ferroptosis that involves lipid peroxidation. Moreover, consistent with our observation of enhanced phagocytosis in APOECh microglia (Figures 1G and 1H), we detected substantial upregulation of phagocytosis-related genes. The differential expression of genes involved in the pathways of lipid metabolism, ferroptosis, phagocytosis, and Aβ binding is shown in the heatmap (Figure 2C).

We validated DEGs enriched in the lipid metabolism and the ferroptosis pathway using qRT-PCR. We detected substantially elevated expression of *ACSL1*, a gene encoding a key enzyme for lipid droplet biogenesis,^45,46^ in APOE3 microglia compared to APOECh microglia when co-cultured with PSEN1 mutant neurons (Figure 2E). In the ferroptosis pathway (Figure 2D), transferrin receptor protein 1 (TFRC) facilitates iron transport into the cell,^47,48^ while SAT1 contributes to lipid peroxidation through the regulation of ALOX15 expression.^49^ Notably, TFRC and SAT1 exhibited increased expression levels in APOE3 microglia compared to APOECh microglia that were co-cultured with PSEN1 mutant neurons (Figure 2F). Conversely, phagocytosis-related genes, such as *ANXA3*,^50^ *CD93*,^51,52^ and *SLC11A1*,^53^ displayed elevated expression levels in APOECh microglia compared to APOE3 microglia when co-cultured with PSEN1 mutant neurons (Figure 2G). These findings suggest that APOECh microglia could exert a protective effect by suppressing lipid droplet formation and ferroptosis but preserving the phagocytosis function.

### APOECh microglia are resistant to Aβ-induced lipid droplet formation, lipid peroxidation, ferroptosis, and impaired phagocytosis

Because PSEN1 E280A mutant neurons produce more Aβ42 than WT neurons,^54^ the elevated expression of *ACSL1* in APOE3 microglia co-cultured with PSEN1 mutant but not WT neurons prompted us to investigate the role of Aβ42 in the induction of *ACSL1* expression. Indeed, treatment with Aβ42 induced *ACSL1* expression in APOE3 but not APOECh microglia (Figure S3A). We did not see a significant difference in the expression of genes associated with disease-associated microglia, such as *ITGAX* and *TREM2*, in APOE3 and APOECh microglia treated with Aβ42 (Figure S3B).

**Figure 3.**
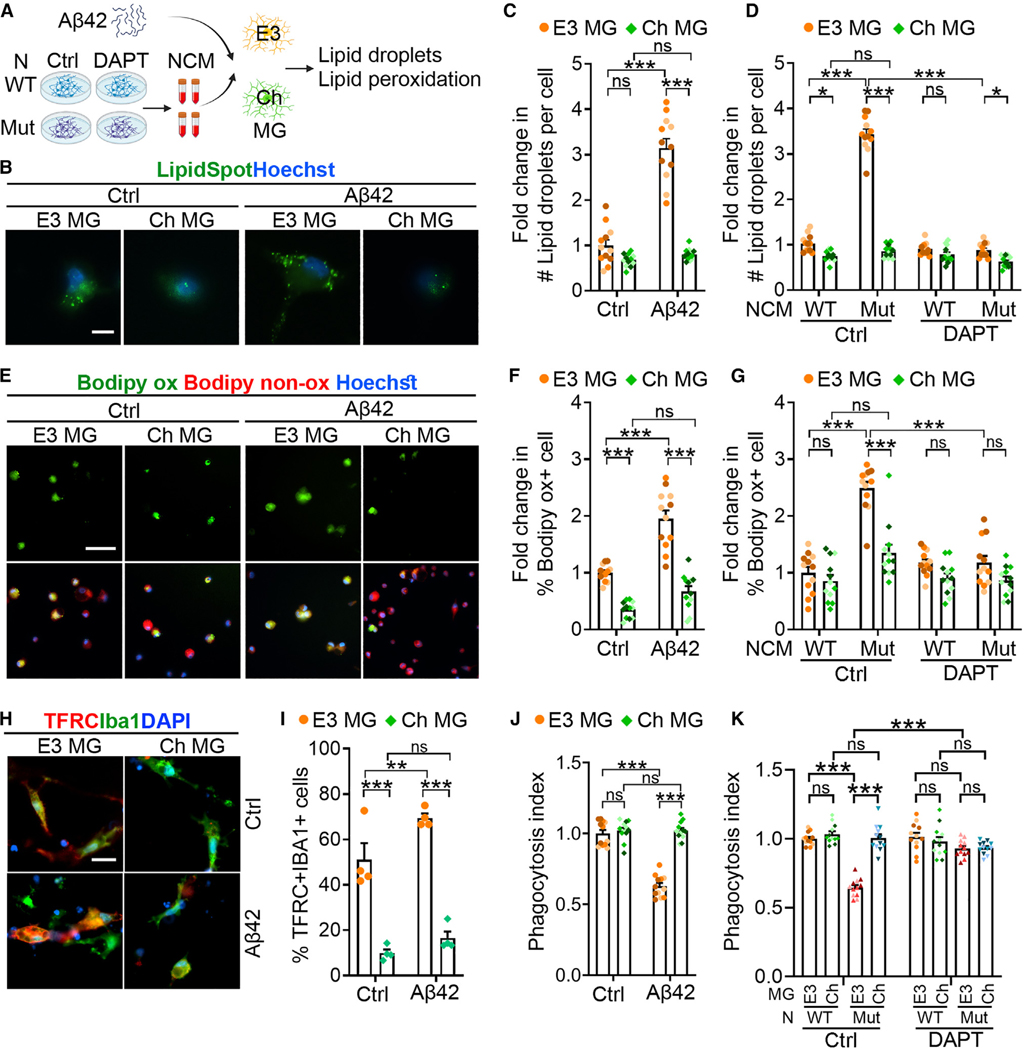
APOECh MG exhibit resistance to Aβ-induced lipid droplet formation, lipid peroxidation, ferroptosis, and impaired phagocytosis (A) A schematic showing the treatment of E3 or Ch MG with Aβ42 or neuronal condition medium (NCM), followed by lipid droplet formation and lipid peroxidation assays. The condition medium was produced from WT or PSEN1 mutant (Mut) neurons treated with vehicle control (Ctrl) or DAPT. (B and C) Aβ42-induced lipid droplet accumulation in E3 but not Ch MG. (B) Representative images of control or Aβ42-treated MG stained with the lipid droplet dye LipidSpot. Scale bar: 10 μm. (C) Quantification of the lipid droplet number (#) per cell. Vehicle-treated control E3 MG was used as the reference for normalization. (D) PSEN1 Mut NCM induced droplet formation in E3 but not Ch MG. Lipid droplet formation in E3 or Ch MG treated with conditioned medium from WT or PSEN1 Mut neurons treated with vehicle control or 10 μM DAPT. E3 MG treated with NCM from control WT neurons was used as the reference for normalization. (E and F) Aβ42 stimulated lipid peroxidation in E3 but not Ch MG. (E) Representative images of E3 or Ch MG treated with vehicle control or Aβ42 and labeled with BODIPY 581/591 C11. Scale bar: 50 μm. (F) Fold change in the percentage of lipid-peroxidized cells (green 488 nm) out of total lipid-positive cells (red 591 nm). Vehicle-treated control E3 MG was used as the reference for normalization.

Next, we examined whether Aβ42 treatment could induce lipid droplet formation in APOE3 and APOECh microglia differentially. We stained Aβ42-treated APOE3 or APOECh microglia with LipidSpot, a dye that stains lipid droplets in cells. Consistent with the *ACSL1* gene expression pattern, we detected substantially increased lipid droplet number in APOE3 but not APOECh microglia (Figures 3A–3C). Similarly, treatment with neuronal conditioned medium (NCM) from PSEN1 mutant neurons induced increased lipid droplet formation in APOE3 microglia compared to treatment with NCM from WT neurons. In contrast, NCM from PSEN1 mutant neurons failed to induce increased lipid droplet formation in APOECh microglia. Addition of NCM from PSEN1 mutant neurons treated with the γ-secretase inhibitor (2S)-N-[(3,5-Difluorophenyl)acetyl]-L-alanyl-2-phenyl]glycine 1,1-dimethylethyl ester (DAPT) failed to induce lipid droplets in APOE3 microglia (Figures 3D and S3C).

Lipid droplets may lead to lipid peroxidation.^55,56^ We next asked whether Aβ42 treatment could induce lipid peroxidation. We stained control or Aβ42-treated APOE3 and APOECh microglia with Bodipy 581/591 C11, a fluorescent dye for lipid peroxidation. Lipid oxidation leads to a change of the fluorescence emission of Bodipy C11 from red (non-oxidized Bodipy) to green (oxidized Bodipy [Bodipy ox]). We found that Aβ42 treatment increased the percentage of Bodipy ox+ cells substantially in APOE3 but not APOECh microglia, compared to vehicle treatment (Figures 3E and 3F). Similarly, treatment with NCM from PSEN1 mutant neurons increased the percentage of Bodipy ox+ cells in APOE3 but not APOECh microglia, compared to treatment with NCM from WT neurons. (Figures 3G and S3D). Furthermore, we detected elevated expression of TFRC, an important regulator of ferroptosis, in Aβ42-treated APOE3 but not APOECh microglia (Figures 3H and 3I).

When treated with Aβ42, APOE3 microglia exhibited reduced phagocytosis, while APOECh microglia preserved their phagocytotic capacity (Figure 3J). The reduced phagocytosis in Aβ42-treated APOE3 microglia was correlated with reduced cell viability, which could be rescued by the treatment with the ferroptosis inhibitor Liproxstatin-1 (Figure S4), suggesting that the reduced phagocytosis is caused by ferroptosis of microglia. Treatment with NCM from PSEN1 mutant neurons also reduced phagocytic activity of APOE3 but not APOECh microglia, while NCM from DAPT-treated PSEN1 mutant neurons did not affect the phagocytosis of APOE3 or APOECh microglia (Figure 3K).

**Figure 4.**
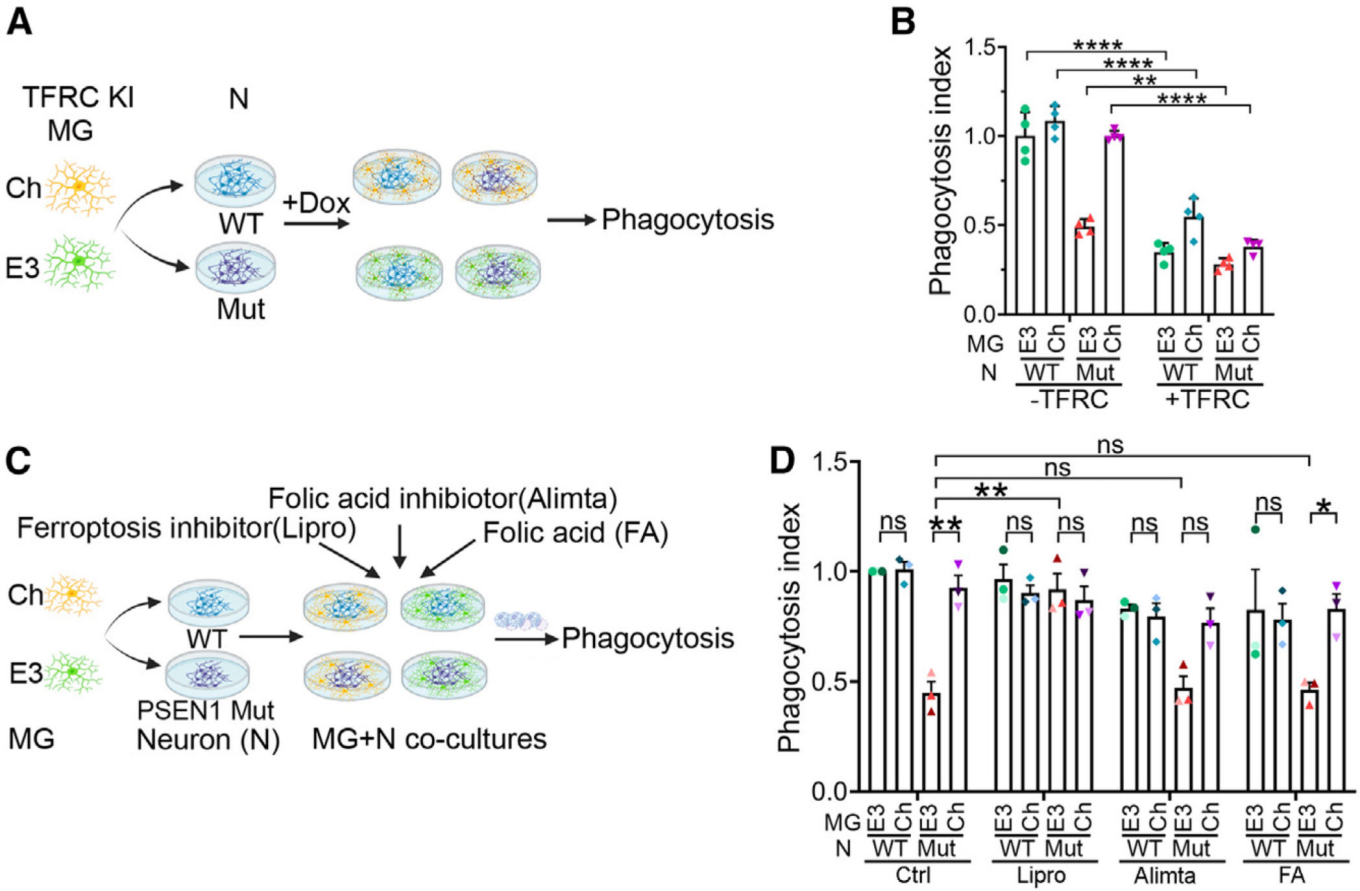
Inhibition of ferroptosis rescues impaired phagocytosis of E3 MG co-cultured with PSEN1 mutant neurons (A) A schematic illustration depicting the co-culture of TFRC knockin (KI) E3 or Ch MG with WT or PSEN1 mutant (Mut) neurons (N) followed by the phagocytosis assay. Doxycycline (Dox) was used to induced TFRC overexpression in TFRC KI MG. (B) Ferroptosis triggered by TFRC reduces MG phagocytosis in N-MG co-cultures. TFRC knockin E3 or Ch MG were co-cultured with WT or PSEN1 Mut neurons with or without Dox induction (− or + TFRC), followed by the phagocytosis assay using pHrodo-labeled human synaptosomes. E3 microglia co-cultured with WT neurons without Dox induction is the reference. Each bar presents four images from one line of iPSC-derived microglia. (C) A schematic illustration showing the co-culture of E3 or Ch MG with WT or PSEN1 mutant (Mut) neurons (N) followed by the treatment of N-MG co-cultures with ferroptosis inhibitor liprostatin-1 (Lipro), folic acid pathway inhibitor (Alimta), or folic acid (FA), and then the phagocytosis assay. (D) Lipro improves MG phagocytosis. E3 or Ch MG were co-cultured with WT or PSEN1 Mut neurons. The co-cultures were subjected to the treatments with vehicle control, 2 μM Lipro, 50 nM Alimta, or 0.2 mg/L FA. The phagocytic index was determined as above, using E3 MG co-cultured with WT neurons and treated with vehicle control as reference for normalization. *n* = 3 lines of iPSC-MG. Error bars are SEM. *p < 0.05, **p < 0.01, ***p < 0.001; ns, *p* > 0.05 by two-way ANOVA followed by Tukey’s multiple comparison test. (G) PSEN1 Mut neurons induced lipid peroxidation in E3 MG. E3 or Ch MG was treated with NCM from WT or PSEN1 Mut neurons treated with vehicle control or 10 μM DAPT, followed by lipid peroxidation assay with BODIPY 581/591 C11 E3 MG treated with NCM from vehicle-treated control WT neurons was used as the reference for normalization. (H and I) Aβ42 induces ferroptosis gene expression in E3 but not Ch MG. (H) Representative images of E3 and Ch MG treated with vehicle control or Aβ42 and stained with anti-TFRC antibody. Scale bar: 20 μm. (I) Quantification of the percentage of TFRC-positive MG. *n* = 4 technical replicate images. Vehicle-treated E3 MG was used as the reference for normalization. (J) Aβ42 decreased phagocytic activity in E3 but not Ch MG. E3 and Ch MG were treated with vehicle or Aβ42. The phagocytosis index was determined by calculating the percentage of pHrodo-positive MG in total MG and then normalizing it using the percentage in vehicle-treated E3 MG as the reference. (K) Blocking Aβ42 production in neurons rescues the phagocytic defect in E3 MG. E3 and Ch MG co-cultured with either WT or PSEN1 Mut neurons were treated with vehicle or DAPT. The phagocytosis index was calculated as described above, using the percentage in vehicle-treated E3 MG co-cultured with WT neurons as the reference for normalization. For (C), (D), (F), (G), (J), and (K), each bar presents a total of 12 images from three lines of iPSC-MG, and each color of dots represents one cell line. Error bars are SEM. ***p < 0.001, **p < 0.01, *p < 0.05; ns, *p* > 0.05 by two-way ANOVA followed by Tukey’s multiple comparison test.

Next, we asked whether the genes in the ferroptosis pathway could affect the phagocytosis. To induce ferroptosis, we overexpressed TFRC in microglia. Specifically, we generated Tet-on inducible *TFRC* knockin (TFRC KI) APOE3 and APOECh microglia (Figure 4A). Induction of TFRC led to substantially reduced phagocytosis in both APOE3 and APOECh microglia co-cultured with WT or PSEN1 mutant neurons (Figure 4B).

To suppress ferroptosis, we treated APOE3 or APOECh microglia co-cultured with WT or PSEN1 mutant neurons with the ferroptosis inhibitor liproxstatin-1. We included vehicle, the folic acid pathway inhibitor Alimta, and folic acid as controls. Treatment of APOE3 microglia co-cultured with PSEN1 mutant neurons with liproxstastin-1 rescued the phagocytic capacity of APOE3 microglia substantially. Neither Alimta nor folic acid exhibited such a rescuing effect (Figure 4D). Treatment of APOECh microglia co-cultured with PSEN1 mutant neurons with liproxstastin-1, Alimta, or folic acid had no significant effect on their phagocytic activity (Figure 4D). These results indicate that ferroptosis affects microglial phagocytosis and reduced ferroptosis is a critical factor for enhanced phagocytosis in APOECh microglia.

### APOECh microglia control pTau levels and maintain electrophysiological activities of PSEN1 mutant neurons

Tau is a soluble microtubule-associated protein responsible for maintaining microtubule stability in neurons. However, under disease conditions, Tau can become abnormally phosphorylated, leading to detachment from microtubules and the formation of Tau oligomer (oTau) and fibrils within the somatodendritic compartment.^57^ This process of Tau oligomerization is believed to be a critical factor for initiating neuronal loss and cognitive impairment observed in neurodegenerative disorders.^58^ We prepared oTau from postmortem brain tissues of patients with AD (Figures S5A and S5B) and introduced them into N-MG co-cultures to serve as a source of the pathological Tau. oTau was applied to N-MG co-cultures for 5 days and the remaining oTau was monitored by immunostaining using an antibody specific to extracellular oTau. There was no significant difference in the amount of residual oTau between APOECh and APOE3 N-MG co-cultures containing WT neurons. However, when oTau was added to the N-MG co-cultures containing PSEN1 mutant neurons, substantially lower residual oTau level was observed in co-cultures with APOECh microglia compared to that in co-cultures with APOE3 microglia (Figures S5C and S5D). These results indicate that APOECh microglia are more effective at clearing oTau compared to APOE3 microglia.

The residual oTau could either stay extracellular or be taken up by neurons. We co-stained neurons with antibodies for pTau and the neuronal marker Tuj1 and quantified the percentage of pTau and Tuj1 double-positive cells. We found a higher percentage of pTau+Tuj1+ cells in N-MG co-cultures with APOE3 microglia than that in N-MG co-cultures with APOECh microglia (Figure S5F, control panel). Moreover, treatment of the N-MG co-cultures with the phagocytosis inhibitor cytochalasin D (cytoD) led to dramatically increased pTau level in neurons co-cultured with either APOE3 or APOECh microglia (Figures S5E and S5F), suggesting that the reduced pTau level in neurons co-cultured with APOECh microglia could be due to the enhanced phagocytosis capacity of APOECh microglia.

Moreover, treatment with oTau enhanced the inflammatory response in APOE3 microglia co-cultured with both WT and PSEN1 mutant neurons, with more dramatic increase in APOE3 microglia co-cultured with PSEN1 mutant neurons. In contrast, APOECh microglia were resistant to oTau-induced inflammatory response (Figure S5G).

**Figure 5.**
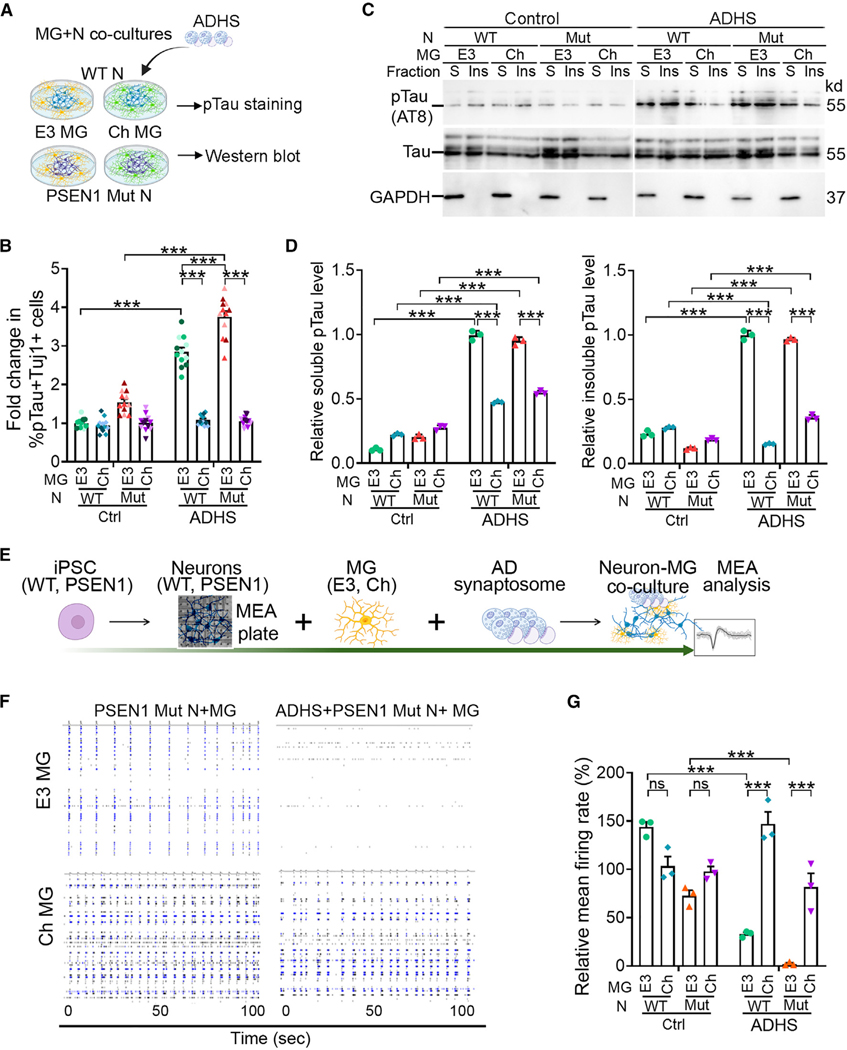
APOECh MG protects neurons from AD insult (A) A schematic illustration of experimental design that co-culture of E3 and Ch MG with WT or PSEN1 mutant (Mut) neurons under the challenge of ADHS, followed by pTau staining and western blot analysis. (B) Fold change of the percentage of pTau (AT8)+Tuj1+ neurons in total Tuj1+ neurons. WT or PSEN1 Mut neurons were co-cultured with E3 or Ch MG. The N-MG co-cultures were treated with vehicle control or ADHS. The percentage of pTau+Tuj1+ neurons in WT neurons co-cultured with E3 MG and treated with vehicle control was used as the reference for normalization. Each bar represents a total of 12 images from three lines of iPSC-MG. (C) Ch MG mitigates ADHS-induced pTau pathology in co-cultured neurons. WT or PSEN1 Mut neurons were co-cultured with E3 or Ch MG and treated with vehicle control or ADHS for 24 h. Subsequently, soluble and insoluble fractions of cell lysates were prepared and subjected to western blot using the pTau (AT8) or total Tau antibody. GAPDH was used as a control. (D) Quantification of western blot results of pTau levels in soluble and insoluble fractions of the N-MG co-cultures treated with vehicle control or ADHS. GAPDH in soluble fractions was used as a loading control for both soluble and insoluble fractions of the same cell lysate. *n* = 3 technical repeats. (E) A schematic of MEA analysis of WT or PSEN1 Mut neurons co-cultured with E3 or Ch MG and treated with ADHS. (F) Representative spike raster plots from MEA analysis of PSEN1 Mut neurons co-cultured with E3 or Ch MG and treated with or without ADHS. MEA assays were recorded over 100 s. (G) Quantification of relative mean firing rate (Hz) from the MEA analysis. PSEN1 Mut neuron-alone cultures were used as the reference. *n* = 3 independent recordings. Error bars are SEM. *p < 0.05, **p < 0.01, ***p < 0.001; ns, *p* > 0.05 by two-way ANOVA followed by Tukey’s multiple comparison test.

Next, we introduced AD human synaptosomes (ADHS)^59^ isolated from postmortem brain tissues of patients with AD (Figures S6A and S6B) into N-MG co-cultures as another source of pathological Tau. Following ADHS challenge, we detected a substantial increase in pTau levels (Figures 5B–5D and S6C), in both soluble form and insoluble form (Figures 5C and 5D), in neurons co-cultured with APOE3 microglia, including WT and PSEN1 mutant neurons. However, the increase of pTau level was mitigated in neurons co-cultured with APOECh microglia (Figures 5B–5D and S6C). An elevation of total Tau level was also detected in neurons when the co-cultures were treated with ADHS, with higher elevation in neurons co-cultured with APOE3 microglia than that in neurons co-cultured with APOECh microglia (Figure S6D). These results indicate that APOECh microglia could protect neurons from pTau accumulation, presumably by preventing the propagation of ADHS-originated pTau across neurons through active phagocytosis.

Neuronal loss and abnormal neural network activity are commonly observed in brains of patients with AD.^60,61^ To determine if APOECh microglia can maintain neural network activity, we co-cultured WT or PSEN1 mutant neurons with APOE3 or APOECh microglia. We challenged the N-MG co-cultures with ADHS and assessed the electrophysiological activity of neurons in the co-cultures using multi-electrode array (MEA), which enables the analysis of neuronal electrophysiological activity at a population scale, including the neuronal network. We first seeded neurons onto MEA plates and measured their neuronal activity as the baseline control. Then, we added APOECh or APOE3 microglia into the neuronal cultures and maintained the N-MG co-cultures for 10 days. Subsequently, we treated the co-cultures with ADHS followed by MEA recording (Figure 5E).

ADHS treatment decreased neural network activity substantially in both WT and PSEN1 mutant neurons co-cultured with APOE3 microglia, with more dramatic decrease in PSEN1 neurons. In contrast, the neural network activity was largely preserved in ADHS-treated neurons (WT and PSEN1 mutant) co-cultured with APOECh microglia (Figures 5F, 5G, S5E, and S5F). These results indicate that APOECh microglia can protect not only PSEN1 mutant neurons but also WT neurons that are subjected to AD insults. ADHS treatment also induced neuroinflammatory response in the co-cultures with APOE3 microglia as revealed by the induction of IL-1β expression, whereas the inflammatory response was much dampened in co-cultures with APOECh microglia (Figure S6F).

### An APOE mimetic peptide mimics APOECh to protect neurons from Aβ or ADHS insult

**Figure 6.**
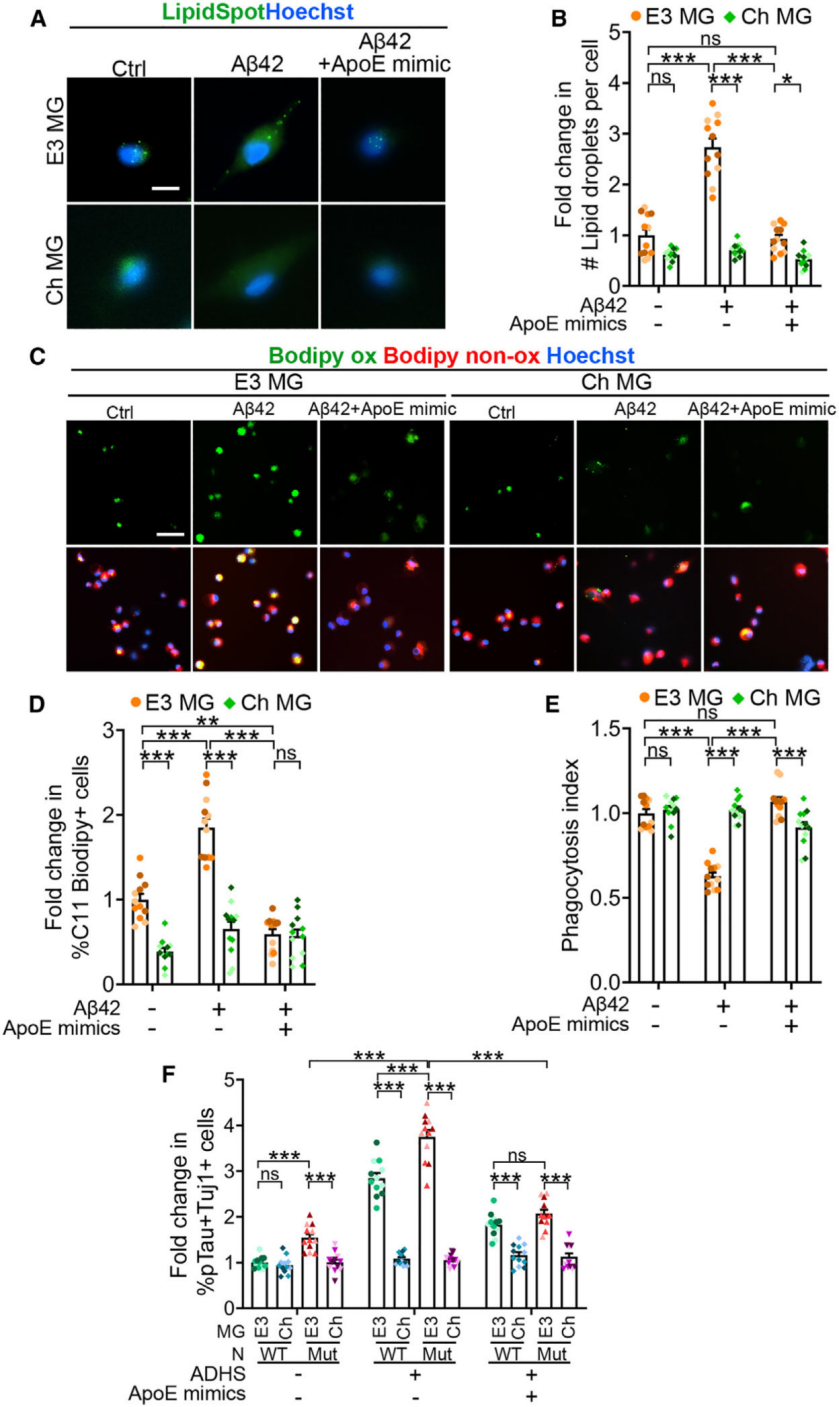
An APOE mimetic peptide protects neurons from AD insults (A and B) An APOE mimetic peptide reduces Aβ-induced lipid droplet accumulation in E3 MG. (A) Representative images of E3 and Ch MG treated with vehicle, Aβ42, or Aβ42 together with the APOE mimetic peptide (APOE mimics) and live stained with LipidSpot and Hoechst. Scale bar: 10 μm. (B) Fold change in the lipid droplet number per cell. Vehicle-treated E3 MG was used as the reference for normalization. (C and D) An APOE mimetic peptide decreases Aβ-induced lipid peroxidation in E3 MG. (C) Representative images of E3 and Ch MG treated with vehicle, Aβ42, or Aβ42 together with the APOE mimetic peptide and labeled with BODIPY 581/591 C11. Scale bar: 10 μm. (D) Fold change in the percentage of lipid-peroxidated cells. Vehicle-treated E3 MG was used as the reference for normalization. (E) The phagocytosis index of E3 or Ch MG treated with vehicle, Aβ42, or Aβ42 together with the APOE mimetic peptide. The phagocytosis index was calculated using the percentage of pHrodo-positive MG and normalized using vehicle-treated E3 MG as the reference. (F) Fold change in the percentage of pTau (AT8)+- Tuj1+ neurons. WT or PSEN1 mutant (Mut) neurons (N) were co-cultured with E3 or Ch MG and then treated with vehicle control, ADHS, or ADHS and the APOE mimetic peptide. The percentage of pTau+Tuj1+ neurons in WT neuron-E3 MG co-cultures treated with vehicle control was used as the reference for normalization. The data from Figure 5B were included here as negative controls for the APOE mimetic peptide treatment. For (B) and (D–F), each bar represents a total of 12 images from three lines of iPSC-MG, and each color of dots represents one cell line. Error bars are SEM. ***p < 0.001, **p < 0.01, *p < 0.05; ns, *p* > 0.05 by two-way ANOVA followed by Tukey’s multiple comparison test.

It has been shown that the protective effect of APOECh is partly due to its reduced affinity for heparan sulfate proteoglycans (HSPG) or other cellular receptors for APOE.^21^ Because an APOE mimetic peptide spanning APOE amino acid residues 133–149 can block the interaction of APOE with its receptors,^62^ we asked whether the APOE mimetic peptide can mimic the APOECh protective effect. We treated APOE3 or APOECh microglia with Aβ42 alone or Aβ42 together with the APOE mimetic peptide and evaluated lipid droplet accumulation in microglia. Remarkably, introduction of the APOE mimetic peptide resulted in a substantial decrease in the lipid droplet number and the level of lipid peroxidation in APOE3 microglia to a level that is comparable to that in APOECh microglia (Figures 6A–6D). Accordingly, the APOE mimetic peptide prevented Aβ-induced impairment of phagocytosis in APOE3 microglia (Figure 6E). Furthermore, the APOE mimetic peptide dramatically reduced the percentage of pTau+Tuj1+ neurons in both WT and PSEN1 mutant neurons treated with ADHS (Figure 6F), indicating the beneficial effects of the APOE mimetic peptide in protecting neurons from pathological insults.

### APOECh microglia reduce pTau level in PSEN1 mutant brain organoids

**Figure 7.**
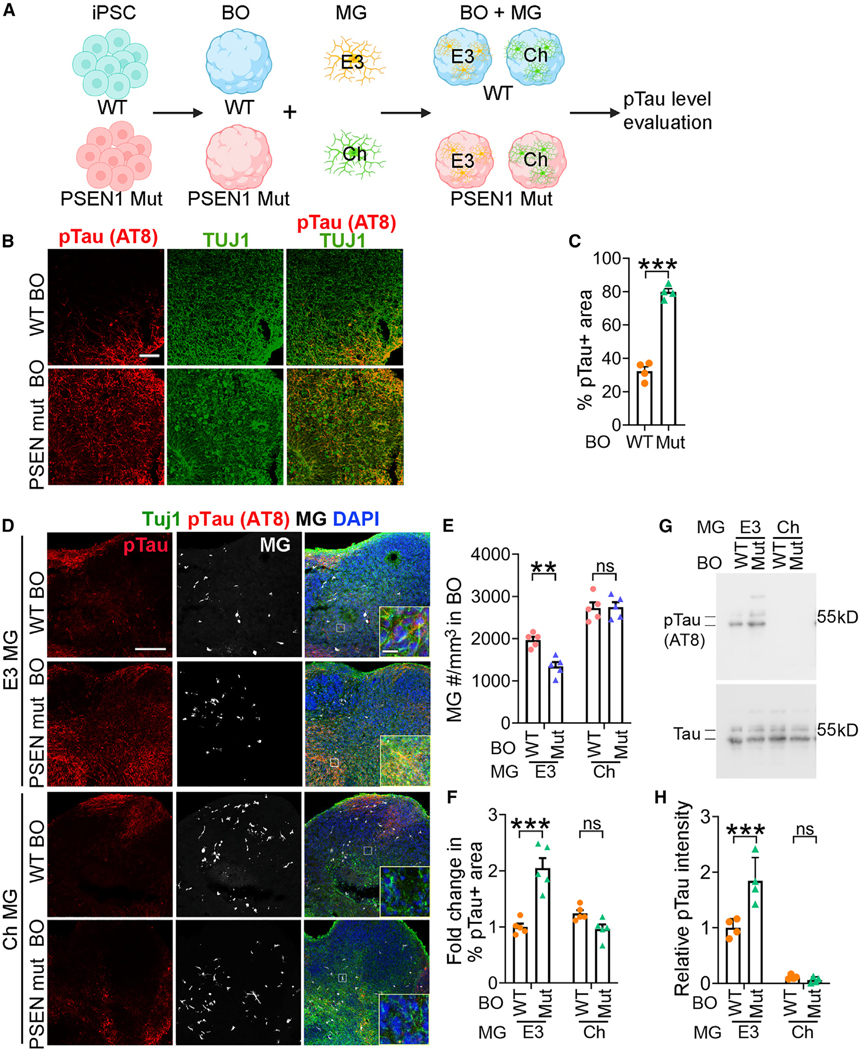
APOECh MG reduces Tau pathology in PSEN1 mutant brain organoids (A) A schematic illustration of brain organoids (BOs) derived from WT or PSEN1 mutant (Mut) iPSCs and infiltrated with E3 and Ch MG, followed by pTau analysis. (B) Representative pTau immunostaining images of WT and PSEN1 Mut BOs at day 70. Scale bar: 10 μm. (C) Quantification of the percentage of pTau+ areas in WT and PSEN1 Mut BOs. *n* = 4 organoids. (D) Representative images of PSEN1 Mut BOs co-cultured with E3 or Ch MG immunostained for pTau (AT8 antibody), the neuronal marker βIII tubulin (TUJ1), and the MG marker IBA1. MG were added to BOs at day 50 of BO differentiation and co-cultured for 20 days. Scale bar: 200 μm. Scale bar for inset images:10 μm. (E and F) Quantification of the MG cell number per mm^3^ (E) and the pTau-positive areas (F) in WT and PSEN1 Mut BOs co-cultured with E3 or Ch MG. *n* = 4 organoids. (G) Western blot of WT or PSEN1 Mut BOs with E3 or Ch MG using antibody for pTau (AT8) or total Tau. (H) Quantification of western blot from (G). The pTau intensity in WT BOs co-cultured with E3 MG was used as the reference for normalization. *n* = 3 technical repeats of brain organoids. Error bars are SEM. **p < 0.01, ***p < 0.001; ns, *p* > 0.05 by two-tailed Student’s t test (C) or two-way ANOVA followed by Tukey’s multiple comparison test (E, F, and H).

Brain organoids derived from hiPSCs provide human cellular models that closely resemble the 3D structure of the human brain and have proven valuable for studying neurodegenerative diseases.^28–30,33,35,63^ To simulate the phenotypes observed in the brains of patients with PSEN1 mutations, we generated brain organoids from both WT and PSEN1 mutant hiPSCs (Figure 7A) following our published protocol with modification.^30^ At day 70 of organoid development, we observed significantly higher levels of pTau in PSEN1 mutant brain organoids compared to WT brain organoids (Figures 7B and 7C).

To mimic the interaction between brain cells and microglia in human brains, we introduced microglia into brain organoids at day 50 of organoid development, a stage when fetal brains are receptive to microglia. 20 days after microglia addition, we evaluated the level of pTau in brain organoids (Figure 7D). We found that APOECh microglia were much more effective in reducing pTau levels in PSEN1 mutant brain organoids than APOE3 microglia (Figures 7D–7H), indicating a protective role of APOECh microglia in PSEN1 mutant brain organoids by ameliorating pTau pathology.

## DISCUSSION

The quest for an effective treatment for AD remains urgent despite considerable efforts in this area. The recent discovery of an association between the presence of an APOECh variant and delayed onset of cognitive impairment reinforces the potential of developing disease-modifying therapies for AD by targeting APOE.^21^

Microglia play a central role in maintaining CNS homeostasis. Many genetic risk factors associated with late-onset AD are highly or specifically expressed in microglia.^64^ Many AD risk genes (i.e., *APOE*, *CLU*, *ABCA7*, *GRN*, *TREM2*, and *CD33*) have been linked to microglia phagocytosis.^65^ Phagocytosis of toxic aggregates by microglia decreases the progression of neurodegenerative disorders, including AD,^64^ while defective phagocytosis could contribute to the risk of these diseases.^66^

In this study, we found that PSEN1 E280A mutant neurons reduced the phagocytic capacity of APOE3 microglia substantially, whereas APOECh microglia are resistant. Moreover, when we added oTau derived from brain tissues from patients with AD to the N-MG co-cultures, we observed substantially less residual oTau in PSEN1 mutant neurons co-cultured with APOECh microglia than in neurons co-cultured with APOE3 microglia. It has been shown that oTau are neurotoxic and can induce neurodegeneration.^58,67,68^ Our results indicate that APOECh microglia are more effective in clearing toxic oTau, presumably due to their resilience to PSEN1 neuron-induced decline in phagocytic capacity. Additionally, we showed that APOECh microglia could help neurons to maintain their neural network integrity when challenged with ADHS.

Our RNA-seq analysis suggests a possible link between lipid metabolism, ferroptosis, and phagocytosis in microglia. By using a ferroptosis inhibitor, we showed that inhibition of ferroptosis is critical for preserving phagocytosis capacity of microglia. We found that APOECh microglia are resistant to Aβ-induced ferroptosis, unlike APOE3 microglia, which succumbed to Aβ-induced ferroptosis. Treatment of APOE3 microglia-PSEN1 mutant neuron co-cultures with a ferroptosis inhibitor rescued the ability of APOE3 microglia to phagocytose synaptosome and clear oTau to a level similar to APOECh microglia, suggesting that the enhanced phagocytic capacity and Tau clearance by APOECh microglia could result from reduced ferroptosis. Consistently, a recent study found that the ferroptosis inhibitor could effectively ameliorate neuronal death and memory loss in a mouse model of AD.^69^

Recent studies have shown that Aβ42 can induce lipid droplet formation and lipid peroxidation in microglia.^70^ Lipid-droplet-accumulating microglia represent a dysfunctional and proinflammatory state in the aging brain.^71^ Additionally, lipid accumulation impairs microglial surveillance of neuronal network activity.^72^ APOE4 has been linked to detrimental effects on lipid droplets in AD microglia through ACSL1.^73^ In this study, we showed that ACSL1 can be induced in APOE3 microglia co-cultured with PSEN1 neurons or treated with the Aβ42 peptide but were not induced in APOECh microglia under the same treatment condition, suggesting that the Aβ42 peptide may be used to study amyloid-induced phenotypes in iPSC-derived cellular models. We have demonstrated the importance of lipid droplet dynamics and lipid peroxidation in microglial phagocytic activity and the involvement of the *ACSL1* gene. Impaired phagocytic activity could lead to an elevated level of pTau and collapse of the neural network. We have shown that APOECh microglia are resistant to Aβ-induced lipid droplet accumulation, lipid peroxidation, and subsequent ferroptosis, thus preserving microglial phagocytic activity, which could in turn prevent pathological Tau propagation and reduce pTau levels in neurons. Our study uncovers an intriguing mechanism underlying the protective effects of APOECh.

It has been shown that the protective effect of APOECh is partly due to its reduced affinity for HSPG or other cellular receptors for APOE.^21^ In this study, we used an APOE mimetic peptide that has been shown to block the interaction of APOE with its receptors^62^ to mimic the APOECh protective effect in APOE3 microglia. We found that the APOE mimetic peptide could reduce the lipid droplet number and the level of lipid peroxidation in APOE3 microglia, to a level comparable to that in APOECh microglia. Furthermore, the APOE mimetic peptide could prevent Aβ-induced impairment of phagocytic capacity in APOE3 microglia and reduce pTau levels in neurons co-cultured with APOE3 microglia and treated with ADHS. Our study provides a strategy that can protect individuals at risk, mimicking the protection provided by APOECh.

Brain organoids have been used to study neurological disorders, including AD pathology.^29,30,35,37^ Generating neuroimmune organoids by adding microglia into brain organoids has allowed us to investigate the effect of APOECh microglia on Tau pathology in a 3D brain-like platform. Consistent with our findings in 2D co-cultures, we observed that APOECh microglia were able to reduce pTau level in PSEN1 mutant brain organoids, presumably by clearing pTau spread or preventing pTau generation.

In summary, we have established 2D and 3D platforms of N-MG co-cultures and neuroimmune organoids to model the protective effect of the APOECh variant. These platforms can be used to model the risk or protective effect of common APOE isoforms, including APOE2, APOE3, and APOE4, and other rare APOE variants, including the APOE3-Jacksonville (V236E) variant and the APOE4-R251G variant.^1,20^ In this study, we have demonstrated that the APOECh variant is causal to neuroprotection, extending the observation made in the 2019 single-patient study^21^ and providing strong rationale for developing potential AD therapies by targeting the protective APOECh variant and using the APOECh variant-carrying cells. Moreover, this study opens up a promising path for screening potential drugs for AD by targeting APOE and its downstream pathways, including suppressing lipid droplet formation, lipid peroxidation, and ferroptosis, and enhancing phagocytosis in microglia. During the revision of this study, two papers characterizing the role of APOECh in mouse models were published.^74,75^

### Limitations of the study

In this study, we have shown that microglial APOE3Ch can protect neurons from Aβ-induced Tau pathology and impaired neural network activity by reducing lipid droplet-mediated ferroptosis and enhancing phagocytosis in microglia using human iPSC-based 2D N-MG co-culture and 3D neuroimmune organoid models. This mechanism of protection remains to be validated in human brain tissues from APOECh carriers. In addition, we have shown that an APOE mimetic peptide can mimic APOECh to protect neurons from Aβ or ADHS insult in N-MG co-cultures. How the APOE peptide exerts its protective effect and whether it acts on neurons, microglia, or the N-MG interface remain to be explored.

## RESOURCE AVAILABILITY

### Lead contact

Further information and requests for resources and reagents should be directed to and will be fulfilled by the lead contact, Dr. Yanhong Shi (yshi@coh.org).

### Materials availability

All unique reagents generated in this study are available from the lead contact upon request with a completed materials transfer agreement.

### Data and code availability

- The RNA-seq datasets generated in this study have been deposited at NCBI GEO and are publicly available as of the date of publication. Accession numbers are listed in the key resources table.
- This paper does not report original code.
- Any additional data that support the findings of this study are available from the lead contact upon reasonable request.

## STAR★METHODS

### EXPERIMENTAL MODEL AND STUDY PARTICIPANT DETAILS

#### Human iPSCs (hiPSCs)

The iPSC line AG14048 (E3/E3) was generated from human fibroblasts (Coriell) (male, age at sampling, 71Y).^42^ The iPSC line ADRC8 (E3/E3, female, age at sampling, 67Y) was generated from human fibroblasts by the UCI ADRC Induced Pluripotent Stem Cell Core from UCI Alzheimer’s Disease Research Center (ADRC) and obtained from Dr. Mathew Blurton-Jones. The iPSC line α was derived from peripheral blood mononuclear cells (PBMC) of patient α (female, age at sampling, 70–71Y) from the Paisa PSEN1 E280A kindred and have the PSEN1 E280A mutation changed to PSEN1 WT by CRISPR/Cas9 editing. Patient α is the carrier of homozygous APOE3Ch and PSEN1 E280A mutation and was reported to have delayed onset of cognitive impairment.^21^ The iPSC line ω was derived from PBMC of patient ω (female, age 70–71 Y) from the Paisa PSEN1 E280A kindred and have the PSEN1 E280A mutation changed to PSEN1 WT by CRISPR/Cas9 editing. Patient ω carries the PSEN1 E280A mutation and developed autosomal dominant AD at the expected age of onset. The iPSC line α and ω were obtained from Drs. Randall Mazzarino and Joseph Arboleda-Velasquez. The iPSC line JIPSC1000APOE3 (male, age at sampling, 55–59Y) and JIPSC1264 APOE Church R136S (male, age at sampling, 55–59Y) were obtained from the Jackson Laboratory.

AG14048 (E3/3) and ADRC8 (E3/3) iPSCs were converted into ECh/Ch iPSCs using CRISPR/Cas9 gene editing.^76,77^ PSEN1 E280A mutant iPSCs were generated from iPSC AG14048 by introducing the PSEN1 E280A mutation using CRISPR/Cas9 editing. We used one clone for each isogenic iPSC line. Table S1 summarizes the clones used in this study.

### METHOD DETAILS

#### Microglia differentiation from hiPSCs

We differentiated microglia from hiPSCs by following a two-stage procedure.^41^ Stage one is the generation of CD43^+^ primitive hematopoietic progenitor cells (HPCs). We used Stem Cell Technologies STEMdiff Hematopoietic Kit (Catalog # 05310) to get HPCs from hiPSCs. Specifically, one day before differentiation, iPSCs were passaged with 0.5 mM EDTA onto 1% Matrigel-coated 6-well plates. Small aggregates of iPSCs were plated evenly at the density of 4–5 aggregates per cm^2^ with medium A. On day 2, 50% medium A was replaced. On day 3, the spent medium was replaced with medium B at 2 mL/well. After that, medium B was supplemented at 1 mL/well every other day. From day 9–11, non-adherent HPCs were collected. Stage two is microglia differentiation. On day 11, HPCs were plated at the density of 10,000 cells per cm^2^ onto 1% Matrigel-coated plates and cultured in microglia differentiation medium (DMEM/F12, 2×insulin-transferrin-selenite, 2×B27, 0.5×N2, 1×Glutamax, 1×NEAA, 400 μM monothioglycerol, and 5 μg/mL insulin). Immediately before use, the microglial differentiation medium was supplemented with a freshly thawed tri-cytokine cocktail (100 ng/mL IL-34, 50 ng/mL TGF-β1, and 25 ng/mL M-CSF). Medium was half changed every other day till day 34. On day 35, cells were resuspended in microglia medium plus 100 ng/mL CD200 and 100 ng/mL CX3CL1 to further mature microglia.

#### Neuron-microglia (N-MG) co-cultures

To set up the N-MG co-cultures, neurons were differentiated from the WT and PSEN1 mutant iPSCs for 21 days.^42^ Microglia were harvested at differentiation day 25 and seeded onto day-21 neuronal cultures at the ratio of N:MG = 5:2. The culture medium was gradually changed from neuronal medium into co-culture medium (Advanced F12, 1×N2, 1×Glutamax, 0.4556 μM 2-mercaptoethanol, 50 ng/mL IL-34), 10 ng/mL M-CSF, 10 ng/mL TGF-β1, 20 ng/mL BDNF and 20 ng/mL GDNF). N-MG co-cultures were maintained for 10 days before analysis. For chemical treatment, vehicle, 1 μM Ferrostatin-1, 50 nM Alimta (pemetrexed),^78^ 1 μM liproxstatin-1, Erastin (5 μM), 500 nM Aβ42, 10 μM DAPT or 0.2 mg/L folic acid were administered to N-MG co-cultures for 24 h followed by subsequent analysis.

#### Neuronal conditioned medium treatment

WT or PSEN neurons were treated with vehicle, 500 nM Aβ42 or 10 μM DAPT for 1 week. Once neuron cultures reached 80% confluence, WT and PSEN neuronal conditioned medium was collected. For medium collection, neurons were first shortly washed with PBS and cultured in complete DMEM F12 medium. After 48 h, neuronal conditioned medium was collected on ice and centrifuged at 9,279g (10,000 rpm) at 4°C for 10 min. Supernatant was collected and stored at −80°C in small aliquots to avoid freeze–thaw cycles. The neuronal conditioned medium was used to treat microglia.

#### Karyotyping analysis

For G-banded karyotyping analysis, 4 wells of iPSCs in a 6-well plate with over 80% confluency were collected. The G-banded karyotyping analysis was performed by the Cytogenetics Core at City of Hope.

#### RNA-seq

Microglia co-cultured with either WT or PSEN1 mutant neurons were purified with immunomagnetic beads before RNA sequencing. Briefly, microglia were co-cultured with neurons for 10 days, the microglia were collected with a gentle blow with 1mL tips to the surface, the suspended media were collected, and the attached cells were singlized with TrypLE. Microglia from the supernatant fraction and the single cell fraction were combined and allowed to bind with CD11b MicroBeads (Miltenyi Biotec, Cat # 130–049-601) following manufacture’s protocol. Total RNA was isolated from the purified microglial cells with TRIzol. RNA quality control was performed by the Integrative Genomics Core at City of Hope.^79^ DEG was determined using the value from APOECh microglia versus that of APOE3 microglia when cocultured with PSEN1 mutant neurons. Heatmap was generated by GraphPad Prism 10 and the images were prepared using Photoshop.

#### Generation of brain organoids

hiPSC-derived brain organoids were generated using an established protocol.^30,79^ Briefly, hiPSCs were maintained in E8 medium (Invitrogen). On day 0 of organoid culture, hiPSCs were dissociated with EDTA, and seeded in suspension in a 6-well plate to form embryoid bodies in E8 medium with 5 μM ROCK inhibitor Y-27632. From day 1 to day 4, cells were cultured in E8 medium without ROCK inhibitor with daily medium change. On day 5, the E8 medium was replaced by the neural induction medium (NIM) containing DMEM-F12 (Invitrogen), 1×N2 supplement (Invitrogen), 1× minimum essential medium NEAA (*MEM*–*NEAA*), and 2 μg/mL Heparin. On day 8, the spheres were embedded in 20–25% Matrigel (Corning) in NIM in a 6-well suspension plate and incubated at 37°C for 4 h, followed by gentle addition of 2 mL of the NIM. On day 10–12, brain organoids were lifted and transferred to a new 6-well plate. NIM was changed daily from day 5 to day 15. On day 15, brain organoids were transferred to a T25 suspension culture flask and cultured in differentiation medium containing DMEM-F12, 1×N2 supplement, 2.5 μg/mL Insulin, 1βGlutamax, 0.5×MEM-NEAA, 3.5 μL/L (V/V) 2-Mercaptoethanol, and 1×B27 supplement on an Orbi-Shaker (Benchmark Scientific) at 50 rpm. Medium was changed every 2–3 days. To stimulate the Aβ production, the brain organoids were extended the medium change to a week before harvest.

#### Co-culture of microglia and brain organoids

Microglia were collected at differentiation day 15 and added to brain organoids at day 50 with a 4×10^5^ cells per organoid. Brain organoids were cultured in U-shaped 96-well plate for overnight after adding microglia. One organoid was maintained in one well. The next day brain organoids were flushed out using pipette and transferred into 25 mL flasks. Brain organoids were maintained on orbital shaker for 20 days in brain organoid medium (DMEM-F12, 1×N2, 1×B27, 1×Glutamax, 0.5×MEM-NEAA, 2.5 μg/mL Insulin, 3.5 μL/L [V/V] 2-Mercaptoethanol) before analysis.

#### Multi-electrode arrays (MEA)

The N-MG cocultures were maintained on 6-well MEA plates (Axion Biosystems). Each well was coated with 1% Matrigel and seeded with 4×10^5^ neurons (and 4×10^4^ mouse astrocytes to serve as the feeder cells), which was used for baseline recording. To investigate the differential impact of E3Ch and E3 microglia on neuronal activity of PSEN1 mutant neurons, microglia were co-cultured with 4×10^5^ neurons (and 4×10^4^ mouse astrocytes) for 10 days. Neuronal activity in the N-MG co-cultures was recorded and compared to the baseline recording of neuronal cultures without microglia. For AD synaptosome treatment, synaptosomes isolated from postmortem brain tissues of patients with AD were introduced into the N-MG cocultures and incubated for 2 days. After that neuronal activity was recorded and compared to the baseline recording. Recording was performed in a Maestro MEA system using the AxIS software (Axion Biosystems). MEA data analysis was performed using the Axion Biosystems NeuralMetric Tool. Synchrony indexes were calculated by NeuralMetric Tool with a synchrony window set as 20 ms.^30,79^

#### Human synaptosome preparation

We performed human synaptosome preparation by following a published protocol.^34^ Human fetal brain tissues at gestation week 21 were obtained from Biosciences Resources. Postmortem brain samples of patients with AD were provided by ADRC of University of Southern California. The brain samples were slowly frozen in 0.32 M sucrose with 10% DMSO and stored at −80°C. To obtain a crude synaptosome fraction, brain tissues were thawed in a 37°C water bath and homogenized in 10 mM Tris buffer (pH 7.4) with proteinase inhibitors (Roche) and phosphatase inhibitors (Sigma-Aldrich) using a glass/Teflon homogenizer (clearance 0.1–0.15 mm). The homogenates were centrifuged at 1,000 g at 4°C for 10 min, then the supernatant was collected and centrifuged again at 10,000 g at 4°C for 20 min. The resultant pellets were suspended in sucrose/Tris solution and stored in a −80°C freezer. Synaptosomes were conjugated to pHrodo-Red dye according to the manufacturer’s protocol.

For synaptosome treatment, we applied synaptosomes from patients with AD to the microglia alone or coculture of neurons and microglia at final concentration of 100 μg/mL for 24 h, followed by MEA analysis, phagocytosis assays, immunostaining, qRT-PCR and western blotting analysis.

#### oTau preparation

oTau was prepared from postmortem brain tissues of patients with AD.^34^ The PBS-soluble fractions of homogenates prepared from patient brain cortex was collected, then tau oligomers were obtained by immunoprecipitation with anti-oligomer Tau antibody T22 (Sigma Aldrich, Cat. ABN454) from the PBS-soluble fractions and validated by western blot analysis. oTau was added into N-MG co-cultures at the concentration of 40 ng/mL and incubated for 5 days before immunostaining. oTau were conjugated to pHrodo-Red dye according to the manufacturer’s protocol.

#### Immunostaining

The adherent cells were fixed with 4% paraformaldehyde for 30 min and washed with PBS. The cells were treated in blocking solution (1×PBS containing 0.1% Triton X-100 and 5% normal donkey serum) for 1 h at RT. After blocking, the samples were incubated with primary antibodies diluted in blocking solution at 4°C overnight, followed by washing and incubation with secondary antibodies. The primary antibodies used were anti-MAP2 (1:6000), anti-Iba1 (1:500), anti-TREM2 (1:500), and anti-oligomer Tau antibody T22 (1:500), and anti-TFRC (1:60). Cells were counterstained with DAPI before mounting. Images were obtained with a Carl Zeiss LSM700 confocal microscope or Nikon Eclipse Ti2 microscope.

For EdU staining (proliferation assay), we followed manufacturer’s instruction (Invitrogen). Briefly, cells were incubated with 10 mM EdU for 5 h, and then fixed with 4%PFA for EdU staining. Then cells were incubated with EdU reaction cocktail for 30 min. After PBS wash, cells were counterstained with DAPI before mounting.

#### Brain organoid sectioning and staining

Brain organoids were fixed with 4% PFA at 4°C overnight and subsequently subjected to 30% sucrose incubation at 4°C overnight. Then organoids were embedded in the OCT compound and sectioned at a thickness of 20 μm using Leica CM3050S. Prior to immunostaining, antigen retrieval was carried out using the following procedure: sections were subjected to microwave heating in citrate buffer (pH 6.0, Cat# C9999, Sigma) followed by cooling to room temperature and then washed 3 times with water. The antigen retrieval procedure was repeated twice and then the sections were ready for immunostaining. Briefly, sections were incubated with blocking solution at RT for 1hr, then incubated with primary antibodies at 4°C overnight, followed by washing and incubation with secondary antibodies. The dilutions of the primary antibodies used for organoid sections were as follows: 1:500 (anti-pTau antibody AT8) and 1:6000 (anti-βIII tubulin antibody Tuj1). Sections were counterstained with DAPI before mounting. Images were obtained with a Carl Zeiss LSM700 confocal microscope or Nikon Eclipse Ti2 microscope.^30,79^

#### Staining with non-antibody probes

Staining with non-antibody probes was performed following the manufacturer’s guidelines. LipidSpot Lipid Droplet Stains (Cat # 70065, Biotium) for lipid droplet staining, BODIPY 581/591 C11 (Cat #D3861, Thermo Fisher Scientific) used as Lipid Peroxidation, Sensor pHrodo Red E. coli BioParticles Conjugate (Cat #P35361, Invitrogen) for Phagocytosis. pHrodo Red.

#### Analysis of protein Solubility by Detergent fractionation

Sequential protein fractionation from differentiated neurons was performed according to published protocols.^80–82^

Briefly, oTau, ADHS or cell pellets were resuspended in RIPA buffer (Thermo fisher Scientific, Cat # 89900) supplemented with 1% Triton X-100, phosphatase (Sigma) and protease (Roche) cocktail inhibitors, incubated on ice for 30 min and, centrifuged at 20,000*g* for 30 min at 4°C. The supernatant was the soluble fraction, and the pellet was washed once with the RIPA buffer, and later added to the total soluble fraction. The pellet was resuspended in 2% SDS lysis buffer [2% (w/v) SDS in 50 mM Tris and 150 mM NaCl, pH 7.6] using 1/3 of the volume used for the Triton-lysis buffer, incubated for 15 min, water sonicated for 5 min and centrifuged at 20,000*g* for 30 min at 20°C. The supernatant was the insoluble fraction.

Protein concentration of each fraction was determined with the Pierce BCA Protein Assay Kit (Thermo), and SDS-PAGE western blot was performed by loading 15 μg of insoluble-fraction (in SDS-DTT loading buffer, DTT) and equal volume of the soluble-fraction onto SDS-PAGE. The remainder of the procedures were performed as described in the western blot section. For quantification, band intensity is shown relative to respective GAPDH in soluble fraction.

### QUANTIFICATION AND STATISTICAL ANALYSIS

#### Statistical analysis

Statistical analysis was performed using GraphPad Prism 8 software. Comparison involving more than two groups was performed using one-way ANOVA followed by Tukey’s post hoc test and corrected *p* values for multiple comparisons are reported. Comparison with more than two groups and comparing to a control group was performed using two-Way ANOVAs followed by Turkey’s multiple comparison post hoc test and corrected *p* values for multiple comparisons are reported. Comparison of two groups was performed using unpaired two-tailed Students t test. The difference is considered significant when *p* < 0.05. The specific statistical analysis for each figure is reported in the figure legends.

## Supplementary Material

1

2

3

## Figures and Tables

**Table:** KEY RESOURCES TABLE

REAGENT or RESOURCE	SOURCE	IDENTIFIER

Antibodies		

Iba1 antibody	Abcam	Cat # ab5076; RRID: AB_2224402
Anti-TREM2 antibody [EPR20243]	Abcam	Cat # ab209814; RRID: AB_3095849
MAP2 antibody - Neuronal Marker	Abcam	Cat # ab5392; RRID: AB_2138153
Phospho-Tau (Ser202, Thr205) Monoclonal Antibody (AT8)	Thermo Fisher Scientific	Cat # MN1020; RRID: AB_223647
Polyclonal Rabbit anti-TUJ1	Covance	Cat # PRB-435P; RRID: AB_291637
Anti-Tau (T22), oligomeric Antibody	Sigma Aldrich	Cat # ABN454; RRID: AB_2888681
Anti-TFRC Antibody	Santa Cruz Biotechnology	Cat # SC-32272; RRID: AB_627167
Anti-Apolipoprotein E Goat pAb	Millipore	Cat # 178479; RRID: AB_10682965

Chemicals, peptides, and recombinant proteins		

ROCK inhibitor Y-27632 dihydrochloride	Reprocell	Cat # 04-0012-10
Retinoic acid	Sigma Aldrich	Cat # R2625
CHIR99021	Cellagen Technology	Cat # C2447-2s
LDN-193189	Cellagen Technology	Cat # C5361-2s
SB431542	Cellagen Technology	Cat # C7243-5
LipidSpot^™^ Lipid Droplet Stains	Biotium	Cat # 70065
BODIPY^™^ 581/591 C11 (Lipid Peroxidation Sensor)	Thermo Fisher Scientific	Cat # D3861
Matrigel	Corning	Cat # 354230
N2 supplement	Life Technologies	Cat # 17502048
B27 supplement	Thermo Fisher Scientific	Cat # 17504044
Essential 8 Medium	Life Technologies	Cat # A1517001
Human Recombinant Insulin solution	Sigma Aldrich	Cat # I9278
MEM NEAA	Thermo Fisher Scientific	Cat # 11140076
DMEM-F12	Gibco	Cat # 11330-032
BrainPhys medium	STEMCELL Technologies	Cat # 05790
Liproxstatin-1 hydrochloride	Tocris Bioscience	Cat # 6113
Ferrostatin-1	MedChemExpress	Cat # HY-100579
Erastin	MedChemExpress	Cat # HY-15763
GDNF	PeproTech	Cat # 450-10
BDNF	PeproTech	Cat # 450-02
Apolipoprotein E/APOE Protein, Human, Recombinant	Sino biological	Cat # 10817-H30E
Dibutyryl-cAMP	Sigma Aldrich	Cat # D0627
STEMdiff^™^ Hematopoietic Kit	STEMCELL Technologies	Cat # 05310
Recombinant Human GM-CSF (carrier-free)	Biolegend	Cat # 572904
Transforming Growth Factor β 1 Human Recombinant TGFB1	Prospec	Cat # CYT-716
Recombinant Human IL-34	PeproTech	Cat # 200-34
ATRA	Sigma Aldrich	Cat # R2625
EGF	PeproTech	Cat # 100-15
FGF	PeproTech	Cat # 100-18B
Trizol	Invitrogen	Cat # 15596018
Gibco GlutaMAX Supplement	Invitrogen	Cat # 35050079
DyNAmo Flash SYBR Green qPCR mix	Thermo Fisher Scientific	Cat # F416
PMSF	Roche	Cat # 837091
pHrodo^™^ Red E. coli BioParticles^™^ Conjugate for Phagocytosis	Invitrogen	Cat # P35361
pHrodo^™^ Red, succinimidyl ester (pHrodo^™^ Red, SE)	Thermo Fisher Scientific	Cat # P36600

Critical commercial assays		

Tetro cDNA synthesis kit	Bioline	Cat # Bio-65043
Amaxa P3 primary 4D-Nucleofector X kit L	Lonza	Cat # V4XP-3024
Stem Cell Technologies STEMdiff^™^ Hematopoietic Kit	STEMCELL Technologies	Cat # 05310

Deposited data		

RNA-seq data of microglia	This study	GSE240609

Experimental models: Cell lines		

Human: AG14048	Coriell Institute	Cat # AG14048
Human: AG14048 APOECh	This study	N/A
Human: ADRC8	UC Irvine	N/A
Human: ADRC8 APOECh	This study	N/A
Human: ADRC8 TFRC knock-in	This study	N/A
Human: ADRC8 APOECh TFRC knock-in	This study	N/A
ω ApoE3	Harvard	N/A
α ApoECh	Harvard	N/A
APOE R136S SNV/SNV “CHRISTCHURCH” (A)	Jackson lab	Cat # JIPSC1264
KOLF2.1J	Jackson lab	Cat # JIPSC1000

Oligonucleotides		

PSEN1 sgRNA	IDT, Coralville	N/A
5′- TAT GCT GGT TGA AAC AGC TC-3′		
PSEN1 ssODN	IDT, Coralville	N/A
5′-GAT TTA GTG GCT GTT TTG TGT CCG AA		
A GGT CCA CTT CGT ATG CTG GTT GAG		
ACC GCT CAG GAG AGA AAT GCA ACG		
CTT TTT CCA GCT CTC ATT TAC TCC T-3′		
APOECh sgRNA	IDT, Coralville	N/A
5′- CCA GAG CAC CGA GGA GCT GC-3′		
APOECh ssODN	IDT, Coralville	N/A
5′-GCG GCG AGG TGC AGG CCA TGC TCG		
GCC AGA GCA CCG AGG AGC TGC GCG TGA		
GCC TCG CCT CCC ACC TGC GCA AGC		
TGC GTA AGC GGC TCC TCC GCG ATG C-3′		
TMEM119-F: 5′- GGA TAG TGG ACT TCT TCC GCC A-3′	IDT, Coralville	N/A
TMEM119-R: 5′- GGA AGG ACG ATG GGT AAT AGG C −3′	IDT, Coralville	N/A
P2RY12-F: 5′- TGC CAA ACT GGG AAC AGG ACC A −3′	IDT, Coralville	N/A
P2RY12-R: 5′- TGG TGG TCT TCT GGT AGC GAT C −3′	IDT, Coralville	N/A
IL1b-F: 5′-GCA GGC CGC GTC AGT TGT TG-3′	IDT, Coralville	N/A
IL1b-R: 5′-CCC GGA GCG TGC AGT TCA GT-3′	IDT, Coralville	N/A
ATG7-F: 5′- CGT TGC CCA CAG CAT CAT CTT C −3′	IDT, Coralville	N/A
ATG7-R:5′ CAC TGA GGT TCA CCA TCC TTG G −3′	IDT, Coralville	N/A
SAT1-F: 5′- CTT TGG AGC CAC CTC TCT ACA G −3′	IDT, Coralville	N/A
SAT1-R: 5′- ACC AGG CTG AAA ATG TCT CTT CC −3′	IDT, Coralville	N/A
TFRC-F: 5′- ATC GGT TGG TGC CAC TGA ATG G −3′	IDT, Coralville	N/A
TFRC-R: 5′- ACA ACA GTG GGC TGG CAG AAA C −3′	IDT, Coralville	N/A
ANXA3-F: 5′- CTC CAC CAG CAG TCT TTG ATG C −3′	IDT, Coralville	N/A
ANXA3-R: 5′- CCT TCA TTT GCC TGC TTG TCC TG −3′	IDT, Coralville	N/A
CD93-F: 5′- GGC AGA CAG TTA CTC CTG GGT T −3′	IDT, Coralville	N/A
CD93-R: 5′- GGA GTT CAA AGC TCT GAG GAT GG-3′	IDT, Coralville	N/A
SLC11A1-F: 5′- CAT CCT CAC GTT CAC CAG CAT G −3′	IDT, Coralville	N/A
SLC11A1-R: 5′- CCA CGA AGT AGA GGT TGA TGG C-3′	IDT, Coralville	N/A
ACSL1-F: 5′- ATCAGGCTGCTCATGGATGACC-3′	IDT, Coralville	N/A
ACSL1-R: 5′- AGTCCAAGAGCCATCGCTTCAG-3′	IDT, Coralville	N/A
ITGAX-F:5′-GATGCTCAGAGATACTTCACGGC-3′	IDT, Coralville	N/A
ITGAX-R: 5′-CCACACCATCACTTCTGCGTTC-3′	IDT, Coralville	N/A
GAPDH-F: 5′- CCT GTT CGA CAG TCA GCC G-3′	IDT, Coralville	N/A
GAPDH-R: 5′-CGA CCA AAT CCG TTG ACT CC-3′	IDT, Coralville	N/A

Recombinant DNA		

AAVS1-TRE3G-TFRC	This study	N/A
pLVX-UbC-rtTA-Ngn2:2A:Ascl1	Addgene	Cat # 127289

Software and algorithms		

ZEN software	Carl Zeiss	https://www.zeiss.com/microscopy/us/products/microscope-software/zen.html
Image-Pro Premier 9.1	Media Cybernetics	http://www.mediacy.com/support/imagepropremier
AxIS software	Axion Biosystems	https://www.axionbiosystems.com/products/axis-software
AxIS Metrics Tool	Axion Biosystems	https://www.axionbiosystems.com/products/axis-software
NeuralMetric Tool	Axion Biosystems	https://www.axionbiosystems.com/products/axis-software
ClustVis	N/A	https://biit.cs.ut.ee/clustvis/
DAVID Bioinformatics Resources 6.8	Laboratory of Human Retrovirology and Immunoinformatics	https://david.ncifcrf.gov/
NIS-Elements AR	Nikon	RRID: SCR_014329
Fiji	Fiji	RRID: SCR_002285
Graphpad Prism 10	Graphpad Software	RRID: SCR_002798

Other		

CytoView MEA 12 plate	Axion BioSystems	Cat # M768-GL1-30Pt200-5
Maestro MEA system	Axion BioSystems	Equipment
Hamamatsu EMCCD	Hamamatsu	Model C9100-13
Orbi-Shaker	Benchmark Scientific	NC0483060

## Highlights

- APOECh microglia can prevent Aβ-induced microglia lipid peroxidation
- APOECh microglia maintain phagocytic activity in response to Aβ42 or PSEN1 neurons
- APOECh microglia protect neurons by reducing pTau and maintaining neural network
- An APOE mimetic peptide can mimic the protective effects of APOECh microglia
