# Supplementary material for: Microglial APOE3 Christchurch protects neurons from Tau pathology in a human iPSC-based model of Alzheimer’s disease
Source: Cell Rep. Author manuscript; Available in PMC 2025 Jan 22. (PMC11753789; doi:10.1016/j.celrep.2024.114982)
Supplement: 1 [file NIHMS2044432-supplement-1.pdf]

Cell Reports, Volume *43*

Supplemental information

Microglial APOE3 Christchurch protects neurons from Tau pathology in a human iPSC-based model of Alzheimer's disease

Guoqiang George Sun, Cheng Wang, Randall C. Mazzarino, Paula Andrea Perez-Corredor, Hayk Davtyan, Mathew Blurton-Jones, Francisco Lopera, Joseph F. Arboleda-Velasquez, and Yanhong Shi

Supplemental Figures and Legends

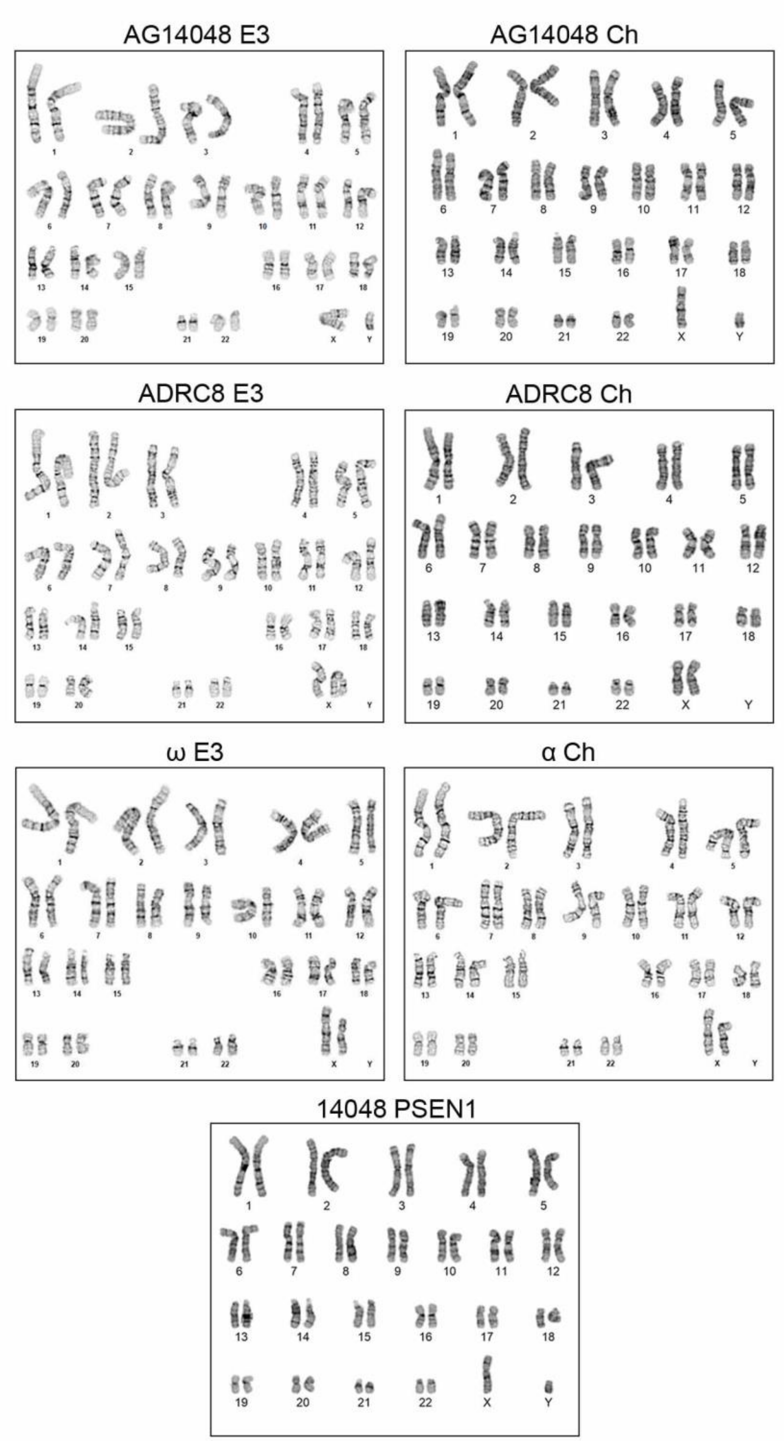

Figure S1. Related to Figure 1. Karyotypes of APOE3 (E3) and APOE3Ch (Ch) iPSC lines and PSEN1 E280A mutant iPSCs. Shown are karyotypes of iPSC 14048 E3, iPSC 14048 Ch, iPSC ADRC8 E3, iPSC ADRC8 Ch, iPSC ω E3, iPSC α Ch, and iPSC 14048 PSEN1 E280A.

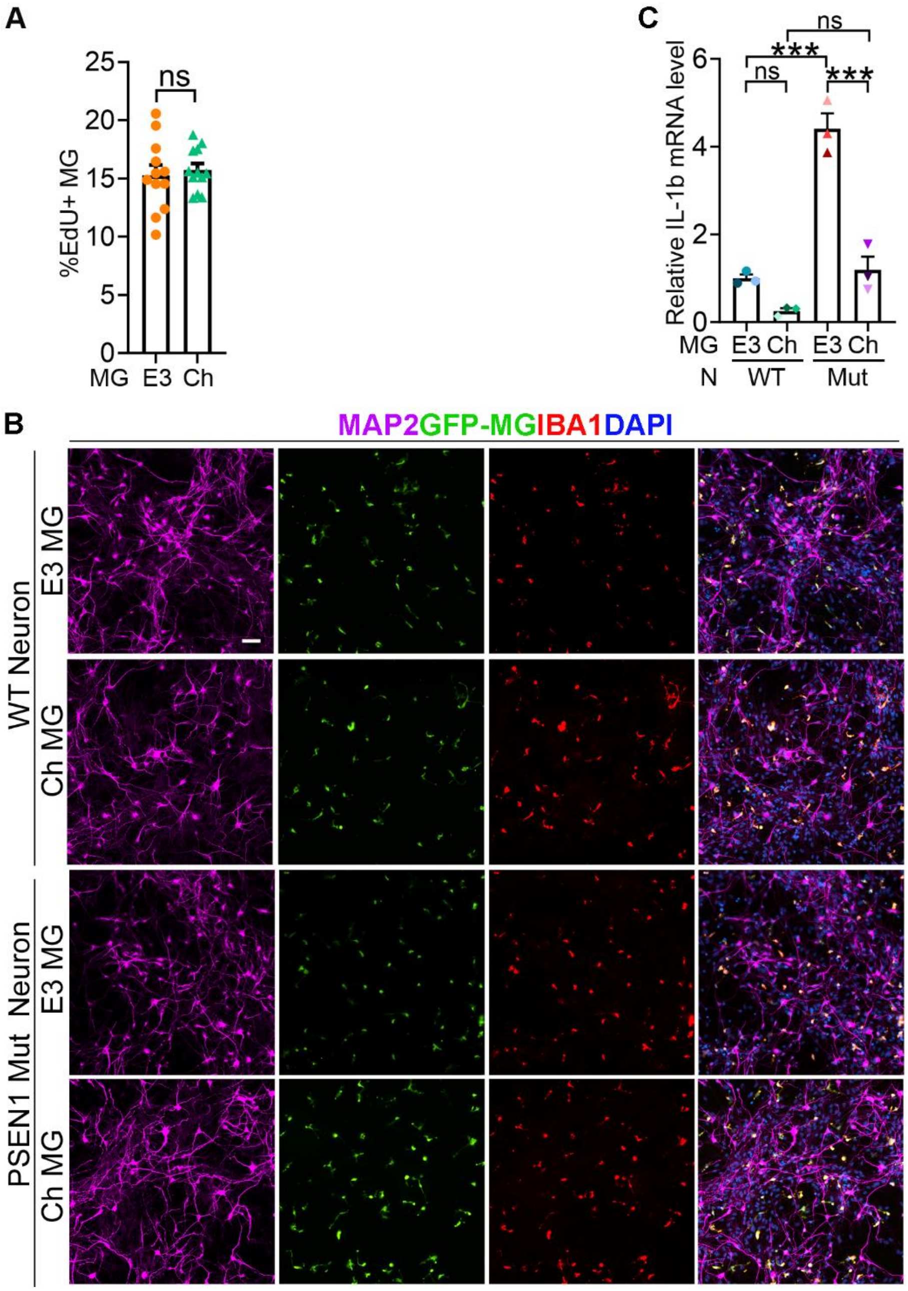

Figure S2. Related to Figure 1. Characterization of APOE3 (E3) and APOE3Ch (Ch) microglia (MG) and neuron (N)-MG co-cultures.

(A) E3 and Ch MG exhibit comparable proliferative rate as revealed by EdU labeling.

(B) Representative images of immunostaining for the neuronal marker MAP2 and the microglia marker IBA1 in N-MG co-cultures. MG were labeled with a GFP reporter. Scale bar: 50 µm.

(C) qRT-PCR analysis of IL-1β gene expression in MG isolated from N-MG co-cultures. The IL-1β level in E3 MG co-cultured with WT neurons was used as the reference for normalization. n=3 biological repeats.

Error bars are SEM of the mean, ***$p < 0.001$ and ns: $p > 0.05$ by two-way ANOVA followed by Turkey's multiple comparison test.

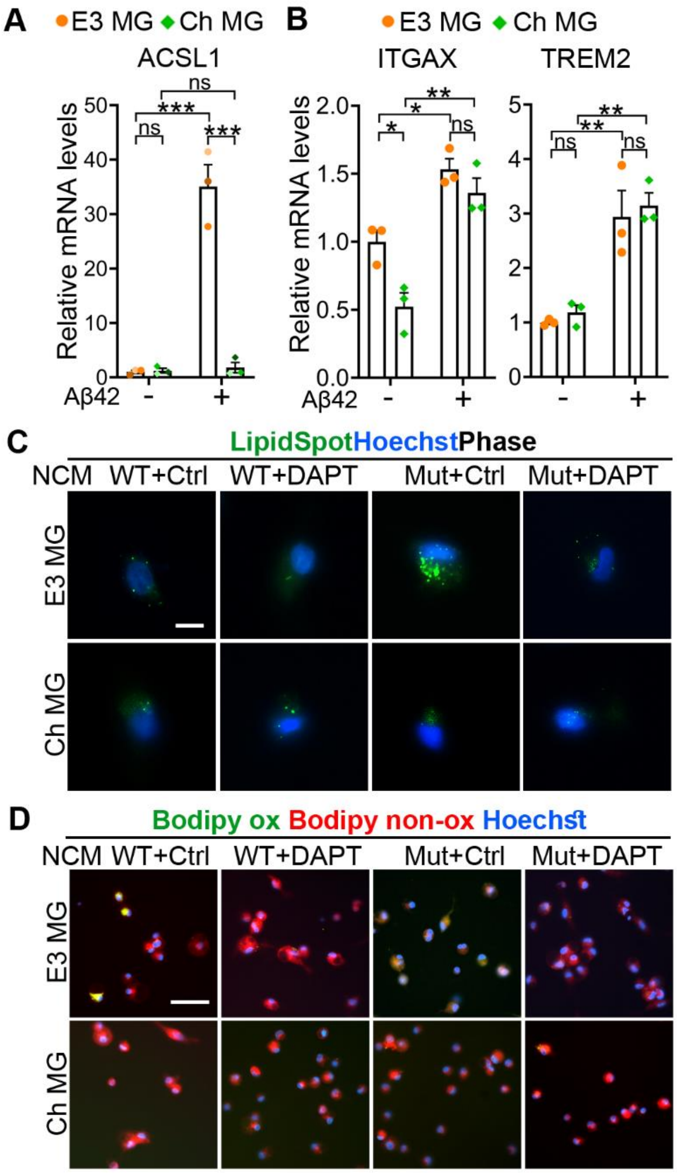

Figure S3. Related to Figure 3. APOECh (Ch) microglia (MG) exhibit resistance to Aβ-induced detrimental effects.

(A) qRT-PCR analysis of ACSL1 expression in Aβ-treated E3 vs Ch MG. n=3 biological repeats.

(B) qRT-PCR analysis of disease-associated microglia genes TREM2 and ITGAX in Aβ-treated E3 vs Ch MG. n=3 biological repeats.

(C) Representative LipidSpot staining images from the lipid droplet assay. E3 or Ch MG were treated with neuronal conditioned medium (NCM) from WT or PSEN1 neurons treated with vehicle control (Ctrl) or DAPT. Scale bar: 10 µm.

(D) Representative images of BODIPY™ 581/591 C11 from the lipid peroxidation assay. E3 or Ch MG were treated with NCM from WT or PSEN1 neurons treated with Ctrl or DAPT. Scale bar: 50 µm.

Error bars are SEM of the mean. *p < 0.05, **p < 0.01, ***p < 0.001, and ns: p > 0.05 by two-way ANOVA followed by Turkey's multiple comparison test.

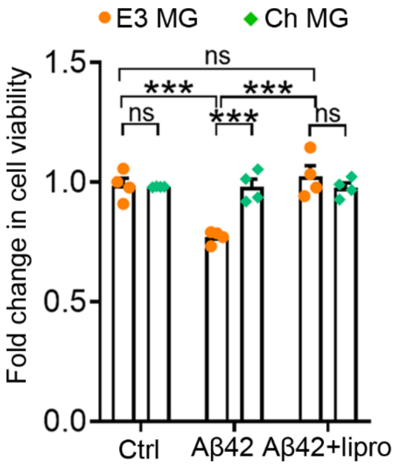

Figure S4. Related to Figure 3. Aβ42 induced cell ferroptosis in APOE3 (E3) but not APOECh (Ch) microglia (MG).

Fold change in cell viability in E3 or Ch MG treated with vehicle, Aβ42, or Aβ42 together with Liproxstatin-1. n=4 technical replicates. Vehicle-treated E3 MG was used as the reference for normalization.

Error bars are SEM of the mean. ***$p < 0.001$ and ns: $p > 0.05$ by two-way ANOVA followed by Turkey's multiple comparison test.

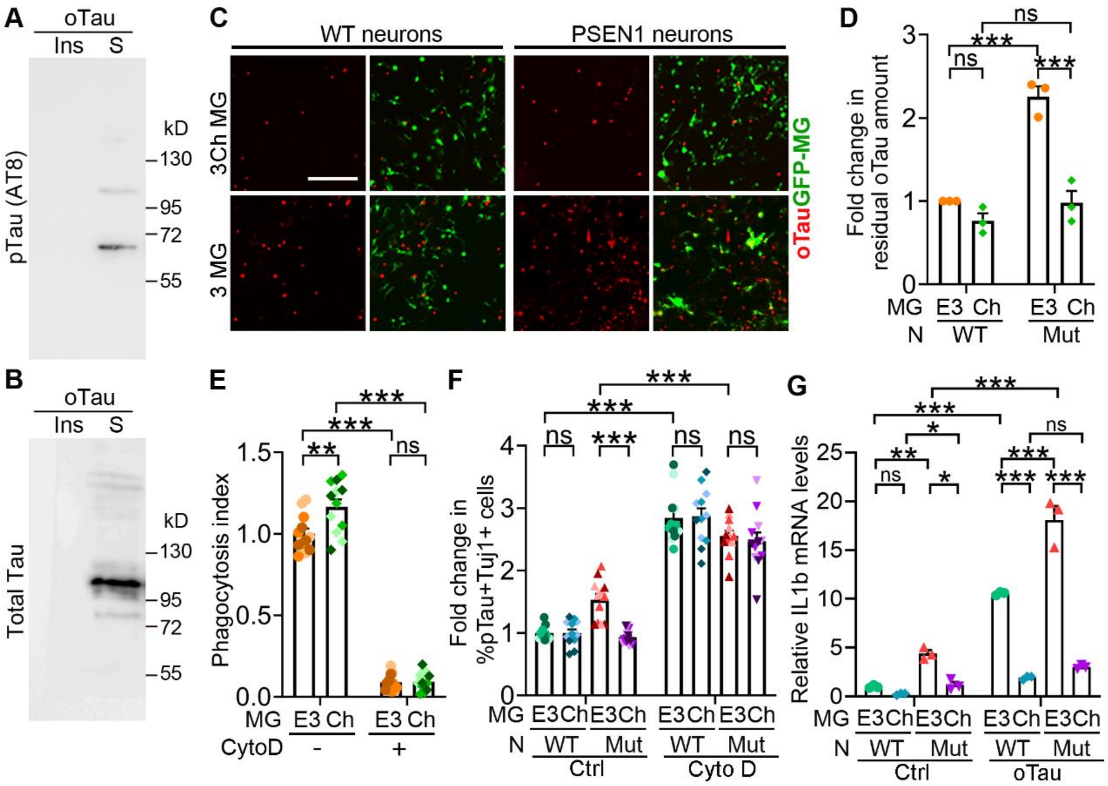

Figure S5. Related to Figure 5. APOECh (Ch) microglia (MG) preserve phagocytotic function when co-cultured with PSEN1 mutant neurons (N) and treated with oTau.

(A, B) Western blot analysis of soluble (s) and insoluble (ins) fractions of oTau for p-Tau (A) and total Tau (B).

(C) Representative images of immunostaining for residual oTau in N-MG co-cultures. Scale bar: 50 µm.

(D) Fold change in residual oTau amount in N-MG co-cultures with E3 or Ch MG. The residual oTau was defined as the positive staining signal of oTau outside of GFP-labeled MG (GFP-MG). The fold change of residual oTau was calculated relative to the amount of residual oTau in WT N-E3 MG co-cultures. n=3 lines of iPSC-derived MG.

(E) Cytochalasin D (CytoD) inhibits phagocytic activity in MG. E3 or Ch MG were treated with 10 µM CytoD. The phagocytic index in E3 MG treated with vehicle control was used as the reference for normalization. Each bar presents a total of 12 images from 3 lines of iPSC-derived MG. Each color of dots represents the data from one MG cell line.

(F) Fold change in the percentage of pTau (AT8)-positive neurons in N-MG co-cultures. The N-MG co-cultures were treated with vehicle control or 10 µM CytoD for 24 hr. The percentage of $pTau^{+}Tuj1^{+}$ neurons in WT N-E3 MG co-cultures treated with vehicle was used as the reference for normalization. Each bar presents a total of 12 images from 3 lines of iPSC-derived MG co-cultured with one line of neurons. Each color of dots represents the data from one MG cell line.

(G) qRT-PCR analysis of IL-1β gene expression in MG isolated from N-MG co-cultures treated with vehicle control or oTau. The IL-1β level in WT N-E3 MG co-cultures treated with vehicle control was used as the reference for normalization. n=3 biological repeats.

Error bars are SEM of the mean. **p < 0.01, ***p < 0.001, and ns: p > 0.05 by two-way ANOVA followed by Turkey's multiple comparison test.

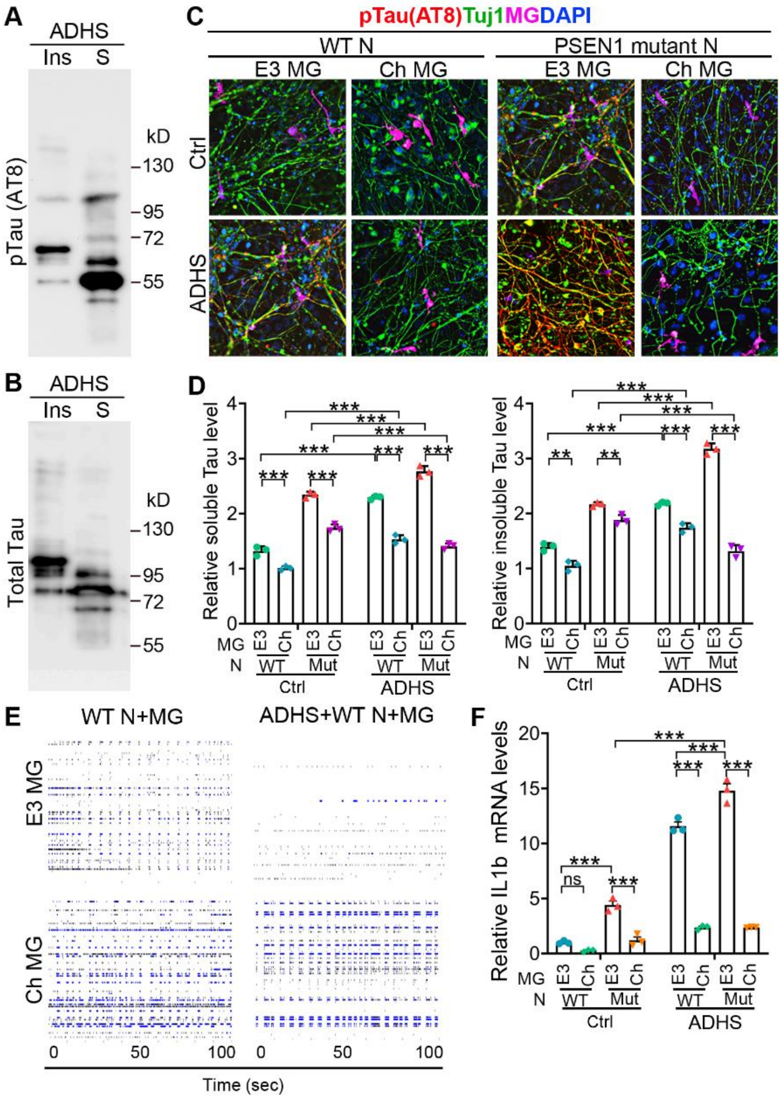

Figure S6. Related to Figure 5. APOECh (Ch) microglia (MG) protect neurons from ADHS treatment.

(A, B) Western blot analysis of soluble (s) and insoluble (ins) fractions of ADHS for pTau (A) and total Tau (B).

(C) Representative images of immunostaining for pTau and Tuj1 in N-MG co-cultures treated with vehicle control (Ctrl) or ADHS. Scale bar: 20 µm.

(D) Quantification of western blot results of total Tau in soluble and insoluble fractions of the N-MG co-cultures treated with Ctrl or ADHS. GAPDH in the soluble fraction was used as a reference. n=3 technical repeats.

(E) Representative spike raster plots from MEA analysis of WT neurons, WT neurons + MG, or WT neurons + MG + ADHS. The MEA signal was recorded over 100 seconds.

(F) qRT-PCR analysis of IL-1β expression in MG isolated from N-MG co-cultures treated with vehicle control or ADHS. The IL-1β level in WT N-E3 MG co-cultures treated with vehicle was used as the reference for normalization. n=3 biological repeats.

Error bars are SEM of the mean. $*p < 0.05$, $**p < 0.01$, $***p < 0.001$, and ns: $p > 0.05$ by two-way ANOVA followed by Turkey's multiple comparison test.

Table S1 Cell line information

iPSC lines	Cell Source	Gender	APOE Genotype	PSEN1 Genotype	Exact Clone
14048 APOE3	Coriell	Male	APOE 3/3	WT	C2
14048 PSEN1 E280A	This study	Male	APOE 3/3	E280A/E280A	F61
14048 APOECh	This study	Male	APOE Ch/Ch	WT	66F
ADRC8 APOE3	UCI	Female	APOE 3/3	WT	C4
ADRC8 APOECh	This study	Female	APOE Ch/Ch	WT	D25
ω APOE3	Harvard	Female	APOE 3/3	WT	B3
α APOECh	Harvard	Female	APOE Ch/Ch	WT	G7
JIPSC1000 APOE3	Jackson lab	Male	APOE 3/3	WT	KOLF2.1J
JIPSC1264 APOE Church R136S	Jackson lab	Male	APOE Ch/Ch	WT	APOE R136S SNV/SNV "Christchurch" (A)

Table S2 Lack of mutation in APOECh sgRNA off-target sites

Table S3 Lack of mutation in PSEN1 sgRNA off-target sites
